# Highly Productive C_3_H_4_/C_3_H_6_ Trace Separation by a Packing Polymorph of a Layered Hybrid Ultramicroporous Material

**DOI:** 10.1021/jacs.3c03505

**Published:** 2023-05-19

**Authors:** Mei-Yan Gao, Andrey A. Bezrukov, Bai-Qiao Song, Meng He, Sousa Javan Nikkhah, Shi-Qiang Wang, Naveen Kumar, Shaza Darwish, Debobroto Sensharma, Chenghua Deng, Jiangnan Li, Lunjie Liu, Rajamani Krishna, Matthias Vandichel, Sihai Yang, Michael J. Zaworotko

**Affiliations:** † Bernal Institute, Department of Chemical Sciences, University of Limerick, Limerick V94 T9PX, Republic of Ireland; ‡ Department of Chemistry, University of Manchester, Manchester, M13 9PL, U.K.; § Van’t Hoff Institute for Molecular Sciences, University of Amsterdam, Science Park 904, 1098 XH Amsterdam, Netherlands; ∥ Institute of Materials Research and Engineering (IMRE), Agency for Science, Technology and Research (A*STAR), 2 Fusionopolis Way 138634, Singapore; ⊥ Department of Materials Science and Engineering, Southern University of Science and Technology, Shenzhen, Guangdong 518055, China

## Abstract

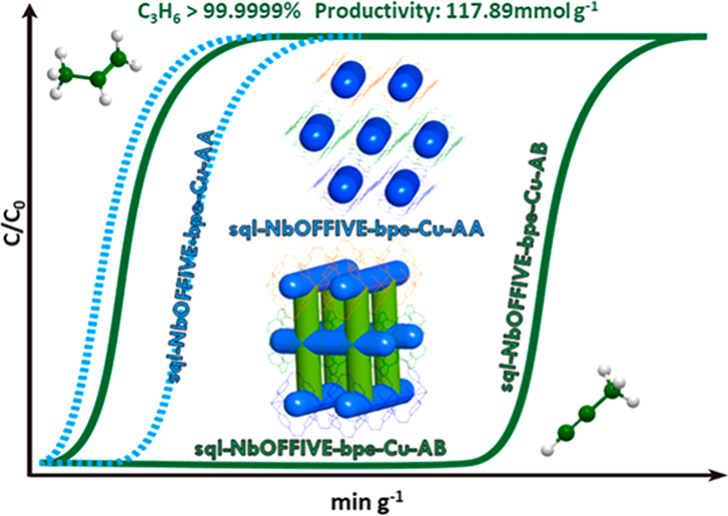

Ultramicroporous materials can be highly effective at trace gas separations when they offer a high density of selective binding sites. Herein, we report that **sql-NbOFFIVE-bpe-Cu**, a new variant of a previously reported ultramicroporous square lattice, **sql**, topology material, **sql-SIFSIX-bpe-Zn**, can exist in two polymorphs. These polymorphs, **sql-NbOFFIVE-bpe-Cu-AA** (**AA**) and **sql-NbOFFIVE-bpe-Cu-AB** (**AB**), exhibit AAAA and ABAB packing of the **sql** layers, respectively. Whereas **NbOFFIVE-bpe-Cu-AA** (**AA**) is isostructural with **sql-SIFSIX-bpe-Zn**, each exhibiting intrinsic 1D channels, **sql-NbOFFIVE-bpe-Cu-AB** (**AB**) has two types of channels, the intrinsic channels and extrinsic channels between the **sql** networks. Gas and temperature induced transformations of the two polymorphs of **sql-NbOFFIVE-bpe-Cu** were investigated by pure gas sorption, single-crystal X-ray diffraction (SCXRD), variable temperature powder X-ray diffraction (VT-PXRD), and synchrotron PXRD. We observed that the extrinsic pore structure of **AB** resulted in properties with potential for selective C_3_H_4_/C_3_H_6_ separation. Subsequent dynamic gas breakthrough measurements revealed exceptional experimental C_3_H_4_/C_3_H_6_ selectivity (270) and a new benchmark for productivity (118 mmol g^–1^) of polymer grade C_3_H_6_ (purity *>*99.99%) from a 1:99 C_3_H_4_/C_3_H_6_ mixture. Structural analysis, gas sorption studies, and gas adsorption kinetics enabled us to determine that a binding “sweet spot” for C_3_H_4_ in the extrinsic pores is behind the benchmark separation performance. Density-functional theory (DFT) calculations and Canonical Monte Carlo (CMC) simulations provided further insight into the binding sites of C_3_H_4_ and C_3_H_6_ molecules within these two hybrid ultramicroporous materials, HUMs. These results highlight, to our knowledge for the first time, how pore engineering through the study of packing polymorphism in layered materials can dramatically change the separation performance of a physisorbent.

## Introduction

Metal–organic materials (MOMs)^1^ such as metal–organic frameworks (MOFs)^2−4^ and porous coordination polymers (PCPs)^5^ are of topical interest because of their potential utility in, for example, gas storage, catalysis, biochemical imaging, and drug delivery.^6−9^ With respect to design, the diversity of their structures and compositions makes MOMs amenable to crystal engineering,^10^ which can enable systematic tuning of composition to control pore size, shape, and chemistry, *i.e*. “pore engineering”.^11−15^ Established approaches to pore engineering include interpenetration, flexibility, open metal sites (OMSs), functionalized ligands, counterion substitution, and pore space partition, which tend to lower pore volume.^15−17^ To our knowledge, pore engineering by different packing of adjacent layers, i.e. polymorphism, has not yet been reported.

**Scheme 1:**
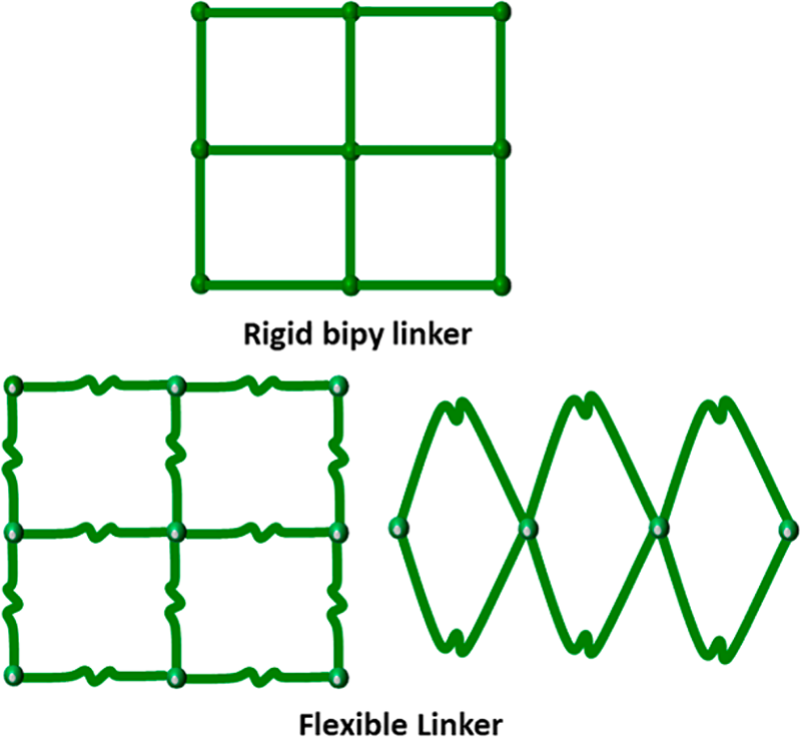
Rigid Linker Ligands Tend to Generate **sql** Topology Coordination Networks (Above) Whereas Flexible Linkers Can Afford Either sql Networks or Spiro-Linked 1D Coordination Polymers (Below)

Hybrid ultramicroporous materials (HUMs), a subclass of MOMs, are based on inorganic pillars such as MFSIX (e.g., GeF_6_^2–^, TiF_6_^2–^, SiF_6_^2–^, SnF_6_^2–^), FOXY (e.g., NbOF_5_^2–^), and M’FFIVE (e.g., AlF_5_^2–^, FeF_5_^2–^). HUMs are of topical interest thanks to their strong and selective binding interactions with small gas molecules including CO_2_ and hydrocarbons (HCs).^18−26,11,20,27−37^ Most HUMs are based on rigid organic linkers (e.g., pyrazine, 4,4′-bipyridine) and pillared by inorganic anions to afford three-dimensional rigid networks. Whether interpenetration of the networks occurs depends mainly on the length and rigidity of linker ligands (Table S2).^18,19,21−23,38−40^ Flexible linker ligands, e.g. 4,4′-dipyridylsulfide (dps), 4,4′-dipyridylsulfone, 4,4′-dipyridylsulfoxide, 1,2-bis(4-pyridyl)ethane (bpe), and 1,3-bis(4-pyridyl)propane (bpp), have the potential to form spiro-linked 1D coordination polymers that, when pillared by inorganic anions, afford 2D square lattice (**sql**) coordination networks (Scheme 1 and Table S3).^24,27,41−44^**sql-SIFSIX-bpe-Zn**, SIFSIX = SiF_6_^2–^, exemplifies such structures and was reported to exhibit high binding affinity for C_2_H_2_ via an induced fit mechanism enabled by flexibility.^27^

**Scheme 2:**
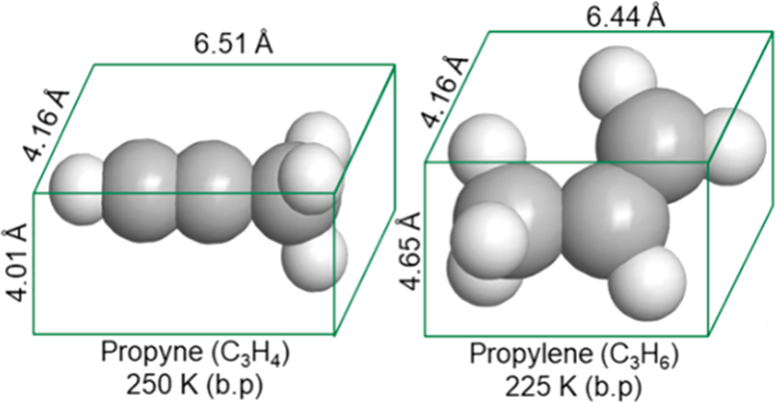
Comparison of the Molecular Structures and Physical Properties of C_3_H_4_ and C_3_H_6_

Herein, we report that solvent-mediated crystallization can result in packing polymorphs of the related material **sql-NbOFFIVE-bpe-Cu**, **sql-NbOFFIVE-bpe-Cu-AA**, **AA**, and **sql-NbOFFIVE-bpe-Cu-AB**, **AB**, which exhibit AAAA and ABAB packing of their **sql** layers, respectively, and study the effect of crystal packing upon the C3 sorption properties of **sql-NbOFFIVE-bpe-Cu**. C3 sorption is relevant because propylene (C_3_H_6_) is a feedstock for the production of commodity chemicals such as acrylonitrile, propylene oxide, and polypropylene.^45−47^ Worldwide propylene production capacity was as high as 140 million tons in 2020, second only to that of ethylene among chemical building blocks.^13^ Further purification of C_3_H_6_ is needed because trace amounts (∼1%) of propyne (C_3_H_4_) must be removed to afford polymer-grade (≥99.95%) C_3_H_6_ for downstream applications.^38,48^ That C_3_H_4_ and C_3_H_6_ exhibit similar physicochemical properties (Scheme 2 and Table S1)^49^ makes it a challenge for porous materials to produce polymer-grade C_3_H_6_. The C3 sorption properties of **AA** and **AB** are addressed through a series of experimental and computational studies.

## Experimental Section

All reagents and solvents were purchased commercially and used as received without further purification, except the precursor CuNbOF_5_·4H_2_O, which was prepared by adapting a reported procedure.^50^

### Synthesis of pcu-NbOFFIVE-bpe-Cu ([Cu(NbOF_5_)(bpe)_2_]_n_)

In a typical reaction, bpe (6.3 mg, 0.035 mmol) in 2 mL of methanol was carefully layered onto CuNbOF_5_·4H_2_O (7 mg, 0.026 mmol) in 2 mL of water. Blue block single crystals were obtained after 4 days in quantitative yield, collected by filtration and washed with methanol three times.

### Synthesis of sql-NbOFFIVE-bpe-Cu-AA-α, AA-α, ([Cu(NbOF_5_)(bpe)_2_]_n_)

In a typical reaction, CuNbOF_5_·4H_2_O (0.0345 g, 0.13 mmol) and bpe (0.0276 g, 0.15 mmol) were added to 11.0 mL of H_2_O/CH_3_OH (v/v = 9:2). The solution was then sealed in a 14.5 mL vial and settled for 1 h. A light blue powder was obtained. This reaction can be readily scaled. When the reaction was conducted at room temperature or 60 °C for 2 months, blue block crystals of **AA-α** were obtained which were suitable for single-crystal X-ray diffraction (SCXRD) testing.

### Preparation of sql-NbOFFIVE-bpe-Cu-AA-β, AA-β

A single crystal of the methanol exchanged phase of **AA-α** was activated at 333 K *in situ* on the goniometer of an SCXRD instrument. After 10 min SCXRD data showed that **AA-α** had transformed to **AA-β**. Bulk samples were prepared through activation at 333 K under vacuum.

### Synthesis of sql-NbOFFIVE-bpe-Cu-AB-α, AB-α, ([Cu(NbOF_5_)(bpe)_2_]_n_)

In a typical reaction, a solution of CuNbOF_5_·4H_2_O (7 mg, 0.026 mmol) in 1 mL of water was carefully layered onto bpe (6.3 mg, 0.035 mmol) in 4 mL of 1,2-dichlorobenzene. Block shaped dark blue single crystals of **AB-α** were obtained after 3 days. The crystals were collected by filtration and washed with methanol three times, yield 85%.

### Preparation of sql-NbOFFIVE-bpe-Cu-AB-β_1_, AB-β_1_

Methanol exchanged **AB-α** was activated by heating at 333 K under vacuum for 12 h and then exposed to air or soaked in water to yield **AB-β**_**1**_.

## Results and Discussion

**Figure 1:**
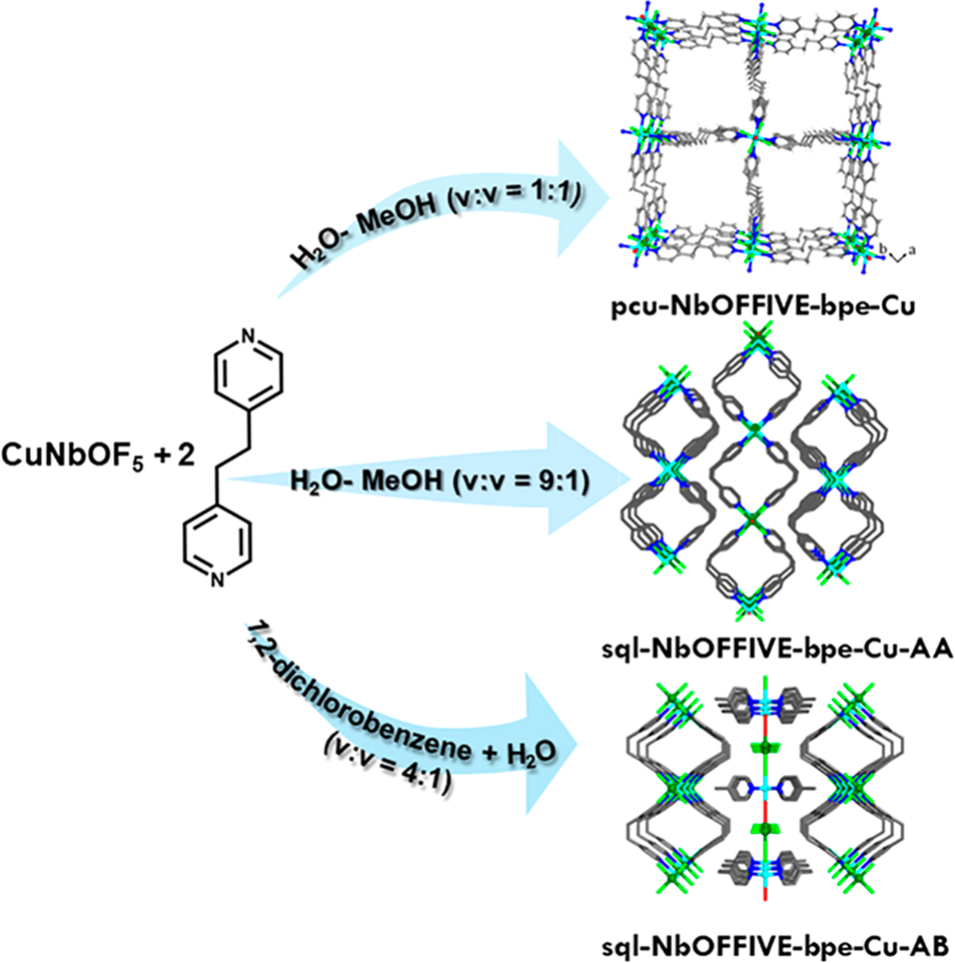
Synthetic conditions used and crystal structures of **pcu-NbOFFIVE-bpe-Cu**, **sql-NbOFFIVE-bpe-Cu-AA-α**, and **sql-NbOFFIVE-bpe-Cu-AB-α**. Color code: turquoise, Cu; green, Nb; red, O; bright green, F; blue, N; gray, C. Hydrogen atoms are omitted for clarity.

Solvent diffusion of CuNbOF_5_·4H_2_O and bpe in various organic solvents and water at room temperature afforded single crystals of three polymorphs of [Cu(NbOF_5_)(bpe)_2_]_n_: a 3D **pcu** network; two 2D **sql** networks. When using 1:1 H_2_O/CH_3_OH (v/v), single crystals of the 3D **pcu** network, **pcu-NbOFFIVE-bpe-Cu**, were obtained with bulk purity (Figures 1 and S12). SCXRD revealed that **pcu-NbOFFIVE-bpe-Cu** had crystallized in the tetragonal space group *P*4/*n* (Table S4). The noninterpenetrated network exhibits ∼9 × 9 Å pores, but crystals of **pcu-NbOFFIVE-bpe-Cu** did not survive guest removal, making it a first generation porous coordination polymer as classified by Kitagawa and co-workers (Figure S3).^5^ When the ratio of H_2_O/CH_3_OH was changed to 9:2 (v/v), single crystals of a 2D **sql** network variant, **AA-α**, were isolated (Figures 1, S1 and S5 upper). SCXRD analysis revealed that **AA-α** had crystallized in the monoclinic space group *C*2/*c* (Table S4). A 4:1 ratio of 1,2-dichlorobenzene and water (v/v) afforded single crystals of a polymorph of the same **sql** network, **AB-α** (Figures 1, S2, and S5 lower). SCXRD analysis revealed that **AB-α** had crystallized in tetragonal space group *P*4_2_/*mmc* (Table S5). Bulk purities of **AA-α** and **AB-α** were confirmed by powder X-ray diffraction (PXRD, Figure S13).

**Figure 2:**
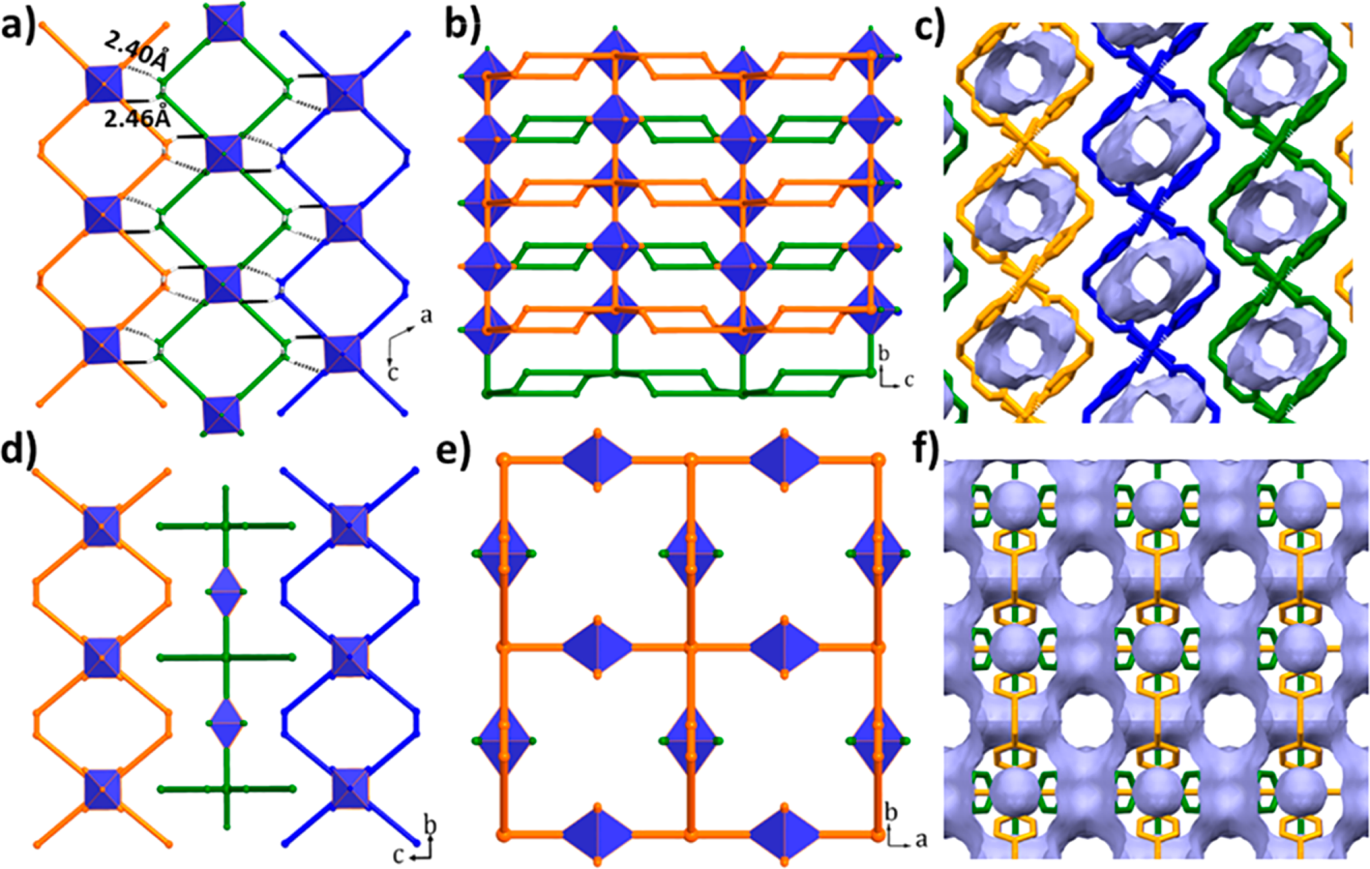
Comparisons of **sql-NbOFFIVE-bpe-Cu-AA-α** (a, b, c) and **sql-NbOFFIVE-bpe-Cu-AB-α** (d, e, f). Simplified crystal structure of **sql-NbOFFIVE-bpe-Cu-AA-α** along the b (a) and a axes (b); (c) 1D channels in **sql-NbOFFIVE-bpe-Cu-AA-α**; Simplified crystal structures of **sql-NbOFFIVE-bpe-Cu-AB-α** along the a (d) and c axes (e); (f) 3D channels in **sql-NbOFFIVE-bpe-Cu-AB-α**. Color code: blue = NbOF_5_; black = hydrogen bonds; Adjacent layers colored orange, green, and blue. Hydrogen atoms omitted for clarity.

Each Cu^2+^ cation in **AA-α** and **AB-α** is six-coordinate, coordinated by four N atoms from bpe ligands as well as one O atom and one F atom from two NbOF_5_^2–^ anions (Figure S4). The pyridyl moieties are oriented in a *gauche* conformation about the C–C single bond backbone, resulting in a V-shaped bis(monodentate) linking mode. The layers in **AB-α** and **AA-α** stack differently. **AA-α** formed an AAAA layer arrangement similar to other **sql** topology HUMs (Figures 2, S5, and S6, some presented using simplified structures).^11,24,27,41−44,51^ Adjacent layers form H-bonds C(bpe ligand)–H···F(NbOF_5_) of 2.40 and 2.46 Å that result in an arrangement reminiscent of a zipper. With respect to **AB-α**, NbOF_5_^2–^ anion pillared Cu(bpe)_2_ chains along the *a*-axis and *b*-axis lie in the *ab*-plane (Figures S5 and S7). **AB-α** was found to exhibit ABAB stacking of layers, resulting in a pore structure distinct from that of **AA-α** and related materials.^27^ The resulting ultramicroporous channels (3.96 × 5.56 Å^2^, after subtracting the van der Waals radii, Figure 2f) represent 29.9% of the unit cell volume as calculated by PLATON.^52^ The 1D channels in **AA-α** resulted in a solvent-accessible space of 24.7% of the unit cell volume (Figure 2c). We anticipated that the inherent flexibility of bpe might enable induced fit or preferential binding toward C_3_H_4_ over C_3_H_6_^10^ and that the electronegative NbOF_5_^2–^ anions lining the channels might preferentially bind alkynes vs alkenes (Figure 2c and 2f).^24,42,53−56^ We therefore undertook a study of the separation performance of these polymorphs toward C_3_H_4_ and C_3_H_6_.

As-synthesized **AB-α** transformed to a narrower-pore phase, **AB-β**, after methanol exchange at 333 K under vacuum for 12 h. We were unable to directly determine the crystal structure of activated (anhydrate) **AB-β**, as it captured water from air at low relative humidity (RH), as revealed by dynamic vapor sorption (DVS) (Figure S26). Figures S27 and S28 reveal that water vapor was adsorbed within minutes at 30% RH, 298 K. The SCXRD structure determined in air, **AB-β**_**1**_, was found to be a hydrate with twisted pores, twisted NbOF_5_^2–^ anions, and undulating pillars, unlike **AB-α** (Figure S9). TGA data collected after holding at 80 °C for 2 h revealed no weight loss, further indicating that **AB-β** is fully activated and that water vapor was captured from the laboratory atmosphere (Figure S18). **AB-β**_**1**_ crystallized in the tetragonal space group *P*4_2_/*mnm* (Table S5). The distance between F atoms (d_*F···F*_) in adjacent pillars changed from 6.33 Å in **AB-α** to 6.91 and 5.37 Å in **AB-β**_**1**_ (Figure S10). Activation of **AA-α** resulted in **AA-β**. Heating at 333 K *in situ* on the SCXRD goniometer enabled structural determination of **AA-β** (Figure S8), which had crystallized in the monoclinic space group *I*2/*m* (Table S4). The *d*_*F···F*_ value decreased from 7.1104 Å in **AA-α** to 6.9260 Å in **AA-β** (Figure S11).

These transformations were also investigated by variable-temperature PXRD (VT-PXRD). **AA-α** converted to **AA-β** by heating at 333 K under N_2_, the PXRD pattern matching that calculated from the SCXRD structure (Figure S16a). Methanol exchanged **AB-α** transformed to desolvated **AB-β** after heating at 393 K under N_2_ (Figure S16b). Methanol exchanged **AB-α** can also transform to **AB-β** by heating at 333 K under vacuum for 6 h (Figure S16c). The partially loaded **AB-β**_**1**_ phase was also observed by VT-PXRD at 333 K, its PXRD diffractogram matching the calculated PXRD pattern of **AB-β**_**1**_. **AB-β** was observed to transform to **AA-β** after heating at 473 K under N_2_ (Figure S16b).

To investigate the porosity of **AA** and **AB**, gas sorption isotherms of CO_2_, at 195 K, and N_2_, at 77 K, were collected (Figure S19). Prior to collection of sorption data, methanol exchanged **AA** and **AB** were activated at 333 K for 12 h under vacuum to generate their respective **β** forms. CO_2_ adsorption by **AB** revealed a stepped isotherm profile with an inflection at low pressure (ca. 0.024 bar) and an uptake of ca. 1.5 mmol g^–1^ after the first step.^57^ A saturated CO_2_ uptake of ∼2.9 mmol g^–1^ corresponds to almost 4 CO_2_ molecules per unit cell. In the case of N_2_ adsorption, an uptake of ∼2.0 mmol g^–1^ was observed. The corresponding values for **AA** revealed CO_2_ and N_2_ uptakes of ∼3.5 mmol g^–1^ and ∼1.0 mmol g^–1^, respectively. Langmuir surface areas of 356 m^2^ g^–1^ for **AA** and 295 m^2^ g^–1^ for **AB** were calculated from 195 K CO_2_ isotherms. The maximum pore size distributions derived from 195 K CO_2_ data were determined to be 3.55 for **AA** and 6.04 Å for **AB**, matching the pore sizes derived from SCXRD data (Figure S19). For a larger probe size, such as N_2_ at 77 K (3.6 Å for N_2_ vs 3.3 Å for CO_2_), **AA** shows lower uptake than that of **AB** because of its narrower pore.

**Figure 3:**
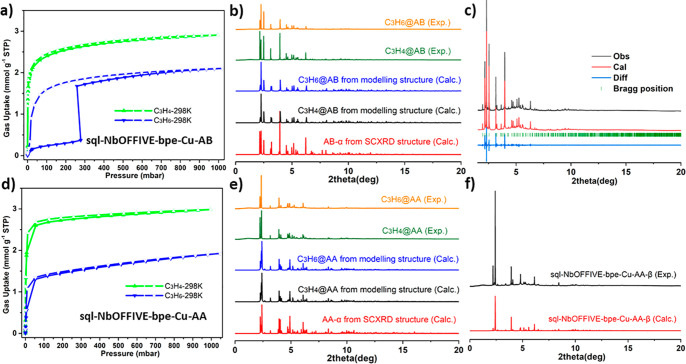
(a) C_3_H_4_ and C_3_H_6_ adsorption isotherms of **sql-NbOFFIVE-bpe-Cu-AB** collected at 298 K; (b) S-PXRD (λ = 0.35424308 Å) patterns of C_3_H_4_-loaded and C_3_H_6_-loaded **sql-NbOFFIVE-bpe-Cu-AB-α** compared with the PXRD patterns calculated from SCXRD and modeling data; (c) Pawley profile fit for **sql-NbOFFIVE-bpe-Cu-AB-β**. The experimental S-PXRD data are presented in black, calculated in red, and the difference between experimental and calculated in blue. Bragg reflections are shown as green bars. Crystal system = Tetragonal, Space group = *P*4_2_/*mnm*, *a* = *b* = 12.4888(4) Å, *c* = 18.8761(6) Å, *V* = 2944.1(2) Å^3^, r_wp = 3.216%, r_exp = 1.821%, r_*p* = 3.127%, GOF = 1.766; (d) C_3_H_4_ and C_3_H_6_ adsorption isotherms of **sql-NbOFFIVE-bpe-Cu-AA** at 298 K; (e) S-PXRD (λ = 0.35424308 Å) patterns of C_3_H_4_-loaded and C_3_H_6_-loaded **sql-NbOFFIVE-bpe-Cu-AA-α** compared with their corresponding PXRD patterns calculated from SCXRD and modeling data; (f) S-PXRD patterns of experimental **sql-NbOFFIVE-bpe-Cu-AA**-**β** compared with calculated PXRD of **sql-NbOFFIVE-bpe-Cu-AA-β** from SCXRD.

Next, we studied the C_3_H_4_ and C_3_H_6_ adsorption properties of **AB** and **AA** at 273 and 298 K (Figures 3a, 3d, S20, and S21). The C_3_H_4_ sorption isotherm of **AB** revealed steep uptake at low pressure and an uptake of 3.04 mmol g^–1^ at 1 bar and 298 K, significantly higher than its C_3_H_6_ uptake (2.10 mmol g^–1^) under the same conditions. We note that the uptake of C_3_H_6_ was negligible at low pressure (0.01 mmol g^–1^ at 0.001 bar; 0.1 mmol g^–1^ at 0.01 bar; 0.23 mmol g^–1^ at 0.1 bar), reflecting the stepped sorption isotherm.^57^ The corresponding C_3_H_4_ uptakes were higher (1.20 mmol g^–1^ at 0.001 bar; 1.93 mmol g^–1^ at 0.01 bar; 2.40 mmol g^–1^ at 0.1 bar, Figure S21). Similar stepped isotherms were reported for GEFSIX-dps-Cu, ELM-12, and ZU-13.^11,58,59^ The uptake ratio of C_3_H_4_/C_3_H_6_ for **AB** at 1 mbar is higher than that of NKMOF-11.^12^ Sample regeneration was realized by exposure to vacuum at 333 K for as little as 10 min. Multiple sorption tests were performed, and similar sorption isotherms were observed, indicating good recyclability (Figure S22).

The differences between the single-component isotherms of C_3_H_4_ and C_3_H_6_ are indicative of potential utility for separation of C_3_H_4_/C_3_H_6_ binary mixtures. In the case of **AA**, the uptake ratio of C_3_H_4_/C_3_H_6_ at 1 mbar (∼3.8) is much lower than that of **AB** (∼120), although **AA** shows a similar uptake of C_3_H_4_ at 1 bar and lower uptake of C_3_H_6_ than **AB** (Figure S21). These results indicate that **AB** offers stronger potential for separation of C_3_H_4_/C_3_H_6_ than **AA**.

Synchrotron PXRD (S-PXRD) data were collected for activated as well as C_3_H_4_ and C_3_H_6_-loaded samples to study the guest-induced structural change. As shown in Figure 3b and 3e, S-PXRD diffractograms of C_3_H_4_ and C_3_H_6_-loaded **AA** and **AB** support the presence of the corresponding **α** phases. The S-PXRD pattern of activated **AA-β** obtained by heating at 333 K under vacuum is consistent with the calculated pattern (Figure 3f). Pawley fitting for **AB-β** revealed tetragonal space group *P*4_2_/*mnm* (Figure 3c) and a unit cell volume of **AB-β** (2944.1(2) Å^3^) slightly smaller than that of **AB-β**_**1**_ (2954.6(4) Å^3^). These results indicate that both C_3_H_4_ and C_3_H_6_ can induce phase changes from the narrow-pore phases (**AA-β** and **AB-β**) to the respective open phases (**AA-α** and **AB-α**).

**Figure 4:**
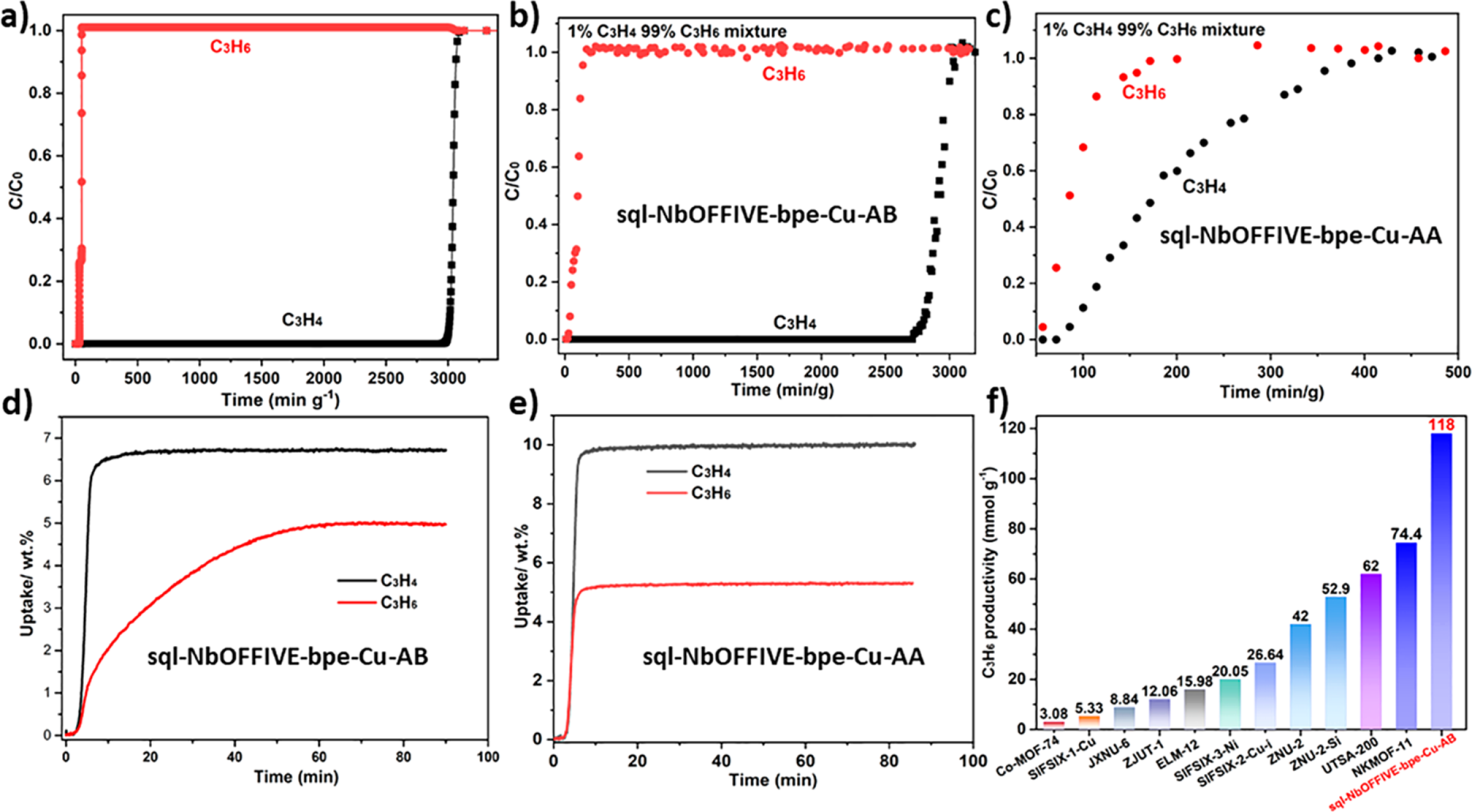
(a) Simulated breakthrough curves of **sql-NbOFFIVE-bpe-Cu-AB** for separation of C_3_H_4_/C_3_H_6_ (1/99) mixture at 298 K; (b) Experimental breakthrough separation of **sql-NbOFFIVE-bpe-Cu-AB** for C_3_H_4_/C_3_H_6_ (1/99) at 298 K (gas velocity: 1.0 cm^3^ min^–1^); (c) Experimental breakthrough separation of **sql-NbOFFIVE-bpe-Cu-AA** for C_3_H_4_/C_3_H_6_ (1/99) at 298 K (gas velocity: 1.0 cm^3^ min^–1^); (d) Gravimetric kinetics of **sql-NbOFFIVE-bpe-Cu-AB** for C_3_H_4_ and C_3_H_6_ uptake (0–1.0 bar) at 303 K; (e) Gravimetric kinetics of **sql-NbOFFIVE-bpe-Cu-AA** for C_3_H_4_ and C_3_H_6_ uptake (1.0 bar) at 303 K; (f) Comparison of C_3_H_6_ productivity in representative benchmark materials for separation of 1/99 C_3_H_4_/C_3_H_6_ mixture.

To quantify the potential of **AB** for separation of the challenging C_3_H_4_/C_3_H_6_ binary mixtures, ideal adsorption solution theory (IAST) calculations were conducted using Dual-site Langmuir–Freundlich (DSLF) and 3-site Langmuir–Freundlich isotherm shape models^60−62^ (Figures S23 and S24, Tables S6 and S7). The calculated adsorption selectivity values for 1:99 and 1:1 C_3_H_4_/C_3_H_6_ binary mixtures are up to 220 and over 180 at 298 K and 1 bar, respectively. Simulated breakthrough data using a methodology described previously predicts excellent separation performance for C_3_H_4_/C_3_H_6_ (Figure 4a).^63−67^

In order to experimentally evaluate the C_3_H_4_/C_3_H_6_ separation performance of **AA** and **AB**, we conducted dynamic column breakthrough (DCB) experiments that mimic typical process conditions with an inlet gas mixture composition of 1:99 (v/v) C_3_H_4_/C_3_H_6_.^12,22^ This C_3_H_4_/C_3_H_6_ gas mixture with a flow rate of 1.0 cm^3^ min^–1^ was passed through a fixed bed column (8 mm diameter) packed with sorbent at 1 bar and 298 K. The fixed beds of methanol exchanged samples were first activated by heating at 353 K in a 20 cm^3^ min^–1^ flow of Helium for about 6 h. DCB experiments were commenced after samples were cooled to room temperature. Gas chromatography (GC) was used to monitor eluted components quantitatively at short sampling intervals (Figure S29; see Supporting Information for the experimental setup). As expected (Figure 4b and 4c), **AB** was indeed found to be more effective for C_3_H_4_/C_3_H_6_ separation than **AA**. For **AB**, C_3_H_6_ breakthrough occurred at 10 min g^–1^, well before C_3_H_4_ (2710 min g^–1^). This represents a C_3_H_4_ uptake capacity (1.2 mmol g^–1^, Table S8) comparable to that of the previous benchmark sorbent, NKMOF-1-Ni (1.21 mmol g^–1^) and Ni@FAU (1.59 mmol g^–1^).^49,68^ During the time lag of 2710 min g^–1^ before breakthrough, GC data showed that the concentration of C_3_H_4_ in the effluent gas stream was <1 ppm (Table S8). According to the DCB profile obtained from a 1/99 mixture, the polymer-grade C_3_H_6_ productivity (>99.99% purity) of **AB** sets a new benchmark value of 118 mmol g^–1^, beyond that of previous benchmark materials (NKMOF-11, 74.4 mmol g^–1^; UTSA-200, 62.0 mmol g^–1^; ZNU-2-Si, 52.9 mmol g^–1^; ZNU-2, 42 mmol g^–1^; SIFSIX-3-Ni, 20.05 mmol g^–1^ and SIFSIX-2-Cu-i, 26.64 mmol g^–1^, Figure 4f).^12,13,38,48,69^ Separation selectivity (α_AC_) was calculated to be 270, exceeding that of reported HUMs with **sql** topology GeFSIX-dps-Cu (82.1), GeFSIX-dps-Zn (65.6).^11^ That **AB** outperformed previous benchmark materials in terms of C_3_H_6_ productivity demonstrates that HUMs can offer both high selectivity and high uptake for challenging gas separations.

We also studied the pure gas adsorption kinetics for C_3_H_4_ and C_3_H_6_ using methanol exchanged samples, whereby activated samples of **AA** and **AB** were exposed to a constant flow of 10 cm^3^ min^–1^ C_3_H_4_ and C_3_H_6_ at 303 K and 1.0 bar. As presented in Figure 4d, the slope of the kinetic curve for **AB** is much steeper for C_3_H_4_ than that of C_3_H_6_, indicating faster adsorption kinetics for C_3_H_4_. The kinetic curves of C_3_H_4_ and C_3_H_6_ level off at 6.8 wt % (1.7 mmol g^–1^) after ca. 10 min and 2.5 wt % (1.2 mmol g^–1^) after ca. 60 min, respectively. Regeneration tests were performed by heating the samples at 353 K under N_2_ flow for ca. 1 h (flow rate: 60 cm^3^ min^–1^), and no changes in uptake were observed after successive cycles (Figure S25). With respect to **AA**, the slope of the kinetic curve is almost the same for C_3_H_4_ and C_3_H_6_, indicating similar adsorption kinetics for C_3_H_4_ and C_3_H_6_ (Figures 4e and S25). That gas adsorption kinetics in **AB** favors C_3_H_4_ over C_3_H_6_ is desirable for efficient gas separation during dynamic DCB tests.

Hydrolytic stability of a sorbent is a prerequisite for utility, prompting us to soak crystals of **AB** in water and perform water vapor sorption experiments using DVS. The water sorption experiment revealed a type I isotherm with approximately 15 wt % uptake at about 90% RH, which is consistent with the weight loss observed in the TGA curve (∼12 wt %, Figures S17 and S26). Cycling tests were performed 10 times and revealed that the sample retained stability when exposed to humidity (Figure S28). Crystals soaked in water for 5 days retained crystallinity (Figure S14).

**Figure 5:**
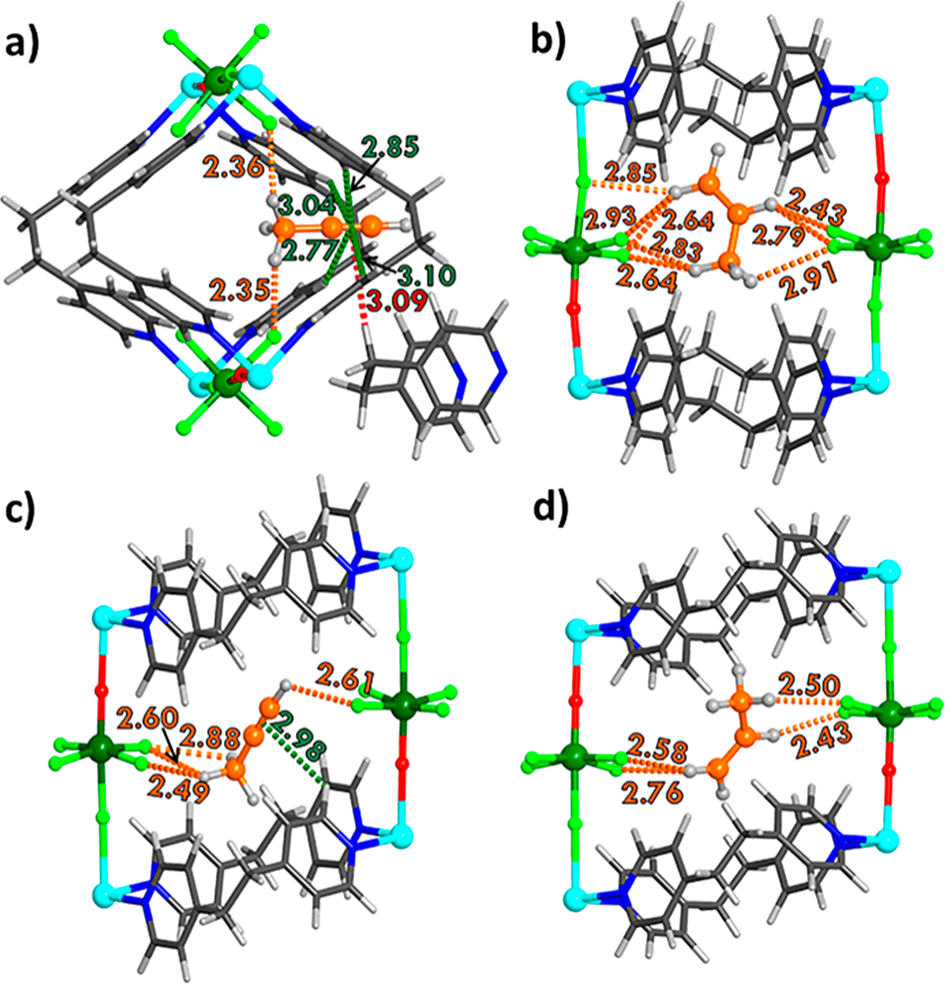
Binding sites of (a) C_3_H_4_ and (b) C_3_H_6_ in **sql-NbOFFIVE-bpe-Cu-AB** (a, b) and **sql-NbOFFIVE-bpe-Cu-AA** (c, d). The closest contacts (Å) between framework atoms and adsorbates are highlighted in orange for C–H···F, green for C–H···π, and red for C–H (with another framework)···π.

Insight into the distinct sorption properties of **AA** and **AB** was gained through DFT calculations, which revealed that **AA** and **AB** have similar lattice energies, the **AB** to **AA** transformation being predicted to be exothermic by −13.2 kJ mol^–1^ per Cu_2_Nb_2_O_2_F_10_(bpe)_4_ formula unit. DFT was also used to identify the most plausible binding sites and their adsorption enthalpies whereas Canonical Monte Carlo (CMC) simulations were conducted to obtain adsorbate occupancy or the density map comprising the binding site regions (see Supporting Information for further details on the computational methodology). For each framework, the energetically most plausible orientations for C_3_H_4_ and C_3_H_6_ are represented in Figure 5. The binding sites were identified using DFT calculations in which atomic positions were optimized so that the binding pockets can adapt to each adsorbate.

The resulting adsorption enthalpies C_3_H_4_ and C_3_H_6_ in **AA** were calculated to be −62.7 (C_3_H_4_) and −65.2 (C_3_H_6_) kJ mol^–1^, respectively. These similar adsorption enthalpies and Gibbs free energy differences are indicative of poor selectivity for C3 hydrocarbons. These values are also in line with experimental data (Figure 4c). In contrast, the adsorption enthalpies of −69.0 (C_3_H_4_) and −53.0 (C_3_H_6_) kJ mol^–1^ calculated for **AB** suggest enhanced binding of C_3_H_4_ and weaker C_3_H_6_ binding compared to **AA**, also in line with experimental observations (Figure 4b). The 16 kJ mol^–1^ difference in adsorption enthalpy for **AB** is also found in the adsorption Gibbs free energy differences Δ*G*_ads_ of −24.4 (C_3_H_4_) and −8.5 (C_3_H_6_) kJ mol^–1^, where a difference of only 4.4 kJ mol^–1^ was calculated for **AA**, with Δ*G*_ads_ values of −19.6 (C_3_H_4_) and −15.0 (C_3_H_6_) kJ mol^–1^, respectively. For both adsorbates, there are hydrogen bonds between H atoms of the adsorbates and framework F atoms (Figure 5), as is typical for HUMs.^70−73^ A detailed analysis of these binding sites, including adsorption energy and distances, is summarized in Table S9. Binding site isosurfaces from CMC simulations are visualized in Tables S11 and S12 and reveal that the density fields, comprising adsorbate mass-middle point occupancies of successful insertion moves, of C_3_H_4_ are larger than those of C_3_H_6_ for all frameworks. This can be attributed to the more linear geometry of C_3_H_4_ resulting in a larger binding area. Furthermore, the CMC results validate the preferred positions of the adsorbates in the framework and its channels in the vicinity of framework F atoms, which can be observed by merging the binding site outcomes from DFT and CMC (Figures S31 and S32). The four modeled crystal structures were used to calculate PXRD patterns, which are a good match for both the experimental S-PXRD data and the PXRD patterns calculated from SCXRD data (Figure 3b, 3e).

## Conclusions

In summary, two packing polymorphs of an **sql** network with intrinsic ultramicropores, **AA** and **AB**, exhibit AAAA and ABAB packing of the **sql** layers, respectively. **AA** is isostructural with **sql-SIFSIX-bpe-Zn** and exhibits intrinsic 1D channels within each **sql** network. **AB** exhibits both intrinsic channels and extrinsic channels between the **sql** networks that arise from the different crystal packing of adjacent layers. Both polymorphs were found to display phase transformations induced by pressure and temperature. **AB** was found to be the most interesting sorbent in terms of sorption properties, as it exhibits excellent C_3_H_4_/C_3_H_6_ separation performance as indicated by a new high for C_3_H_4_/C_3_H_6_ experimental selectivity (270) and a new benchmark for polymer-grade C_3_H_6_ productivity (118 mmol g^–1^) from a 1:99 C_3_H_4_/C_3_H_6_ binary mixture. Modeling studies provided insight into the selective binding sites for C_3_H_4_. This work has not only resulted in a new benchmark for C_3_H_4_ separation from C_3_H_6_ but also brings a new approach to pore engineering. Specifically, whereas the packing polymorphs exhibit similar surface areas, their pore chemistry, size, and shape are distinctly different.

## Supporting Information Available

Experimental and characterization details; additional figures and images; PXRD patterns; VT-PXRD patterns; TGA analysis, DVS analysis, and Sorption isotherms. (PDF)
