# Supplementary material for: Highly Productive C3H4/C3H6 Trace Separation by a Packing Polymorph of a Layered Hybrid Ultramicroporous Material
Source: J Am Chem Soc. 2023 May 19;145(21):11837–45. doi: 10.1021/jacs.3c03505 (PMC10236493; doi:10.1021/jacs.3c03505)
Supplement: Supplementary file 1 — ja3c03505_si_001.pdf [file ja3c03505_si_001.pdf]

## Supplementary Information for

# Highly Productive $C_3H_4/C_3H_6$ Trace Separation by a Packing Polymorph of a Layered Hybrid Ultramicroporous Material

Mei-Yan Gao,[a] Andrey A. Bezrukov,[a] Bai-Qiao Song,[a] Meng He,[b] Sousa Javan Nikkhah,[a] Shi-Qiang Wang,[d] Naveen Kumar,[a] Shaza Darwish,[a] Debobroto Sensharma,[a] Chenghua Deng,[a] Jiangnan Li,[b] Lunjie Liu,[e] Rajamani Krishna,[c] Matthias Vandichel,[a] Sihai Yang,[b] and Michael J. Zaworotko[a]*

[a] Bernal Institute, Department of Chemical Sciences, University of Limerick, Limerick V94 T9PX, Republic of Ireland
E-mail: xtal@ul.ie

[b] Department of Chemistry, University of Manchester, Manchester, M13 9PL, UK

[c] Van't Hoff Institute for Molecular Sciences, University of Amsterdam, Science Park 904, 1098 XH Amsterdam, Netherlands.

[d] Institute of Materials Research and Engineering (IMRE), Agency for Science, Technology and Research (A*STAR), 2 Fusionopolis Way 138634, Singapore

[e] Department of Materials Science and Engineering, Southern University of Science and Technology, Shenzhen, Guangdong, 518055, China.

# Table of Contents

Characterization, Experimental Methods and Sorption Studies .... 3
Physical and Electronic Parameters of Sorbent .... 11
Summary of hybrid ultramicroporous materials (HUMs) .... 11
Tables of Crystal Data .... 13
Optical Image .... 15
Crystal Structures .... 15
PXRD Patterns .... 19
TGA Curves .... 21
Sorption Studies .... 22
Fitting Isotherms .... 24
IAST selectivity .... 25
Fitting Related Parameter .... 25
Experimental Breakthrough Related Data .... 26
Gravimetric Kinetics .... 27
Water Sorption .... 29
Experimental Setup of DCB .... 30
Modeling Studies .... 31
References .... 37

# Characterization, Experimental Methods and Sorption Studies

### 1. Powder X-ray diffraction (PXRD)

Powder X-ray diffraction patterns were recorded on a PANalytical X'Pert MPD Pro (Cu Ka, $\lambda$ = 1.5418 Å) with a 1D X'Celerator strip detector. Experiments were conducted in continuous scanning mode with the goniometer in the theta-theta orientation. Incident beam optics included the Fixed Divergences slit with anti-scatter slit PreFIX module, with a 1/8° divergence slit and a 1/4° anti-scatter slit, as well as a 10 mm fixed incident beam mask and a Soller slit (0.04 rad). Divergent beam optics included a P7.5 anti-scatter slit, a Soller slit (0.04 rad), and a Ni β filter. The data were collected in the range of 2θ = 3-50. Raw data was then evaluated using the X'Pert HighScore Plus™ software V 4.1 (PANalytical, The Netherlands).

### 2. In-situ Variable Temperature Powder X-ray Diffraction (VT-PXRD)

Diffractograms at different temperatures were recorded using a PANalytical X'Pert Pro-MPD diffractometer equipped with a PIXcel3D detector operating in scanning line detector mode with an active length of 4 utilizing 255 channels. Anton Paar TTK 450 stage coupled with the Anton Paar TCU 110 Temperature Control Unit was used to record the variable temperature diffractograms. The diffractometer is outfitted with an Empyrean Cu LFF (long fine-focus) HR (9430 033 7300x) tube operated at 40 kV and 40 mA and Cu Kα radiation ($\lambda_\alpha$ = 1.54056 Å) was used for diffraction experiments. Continuous scanning mode with the goniometer in the theta-theta orientation was used to collect the data. Incident beam optics included the Fixed Divergences slit, with a 1/4° divergence slit and a Soller slit (0.04 rad). Divergent beam optics included a P7.5 anti-scatter slit, a Soller slit (0.04 rad), and a Ni-β filter. In a typical experiment, 20 mg of sample was ground into a fine powder, and was loaded on a zero background sample holder made for Anton Paar TTK 450 chamber. The data was collected from 4°-40° (2θ) with a

step-size of 0.0167113° and a scan time of 50 seconds per step. Crude data were analyzed using the X'Pert HighScore Plus™ software V 4.1 (PANalytical, The Netherlands). The sample was heated up to 453 K under $N_2$ atmosphere.

**3. Thermogravimetric analysis (TGA)**

Thermograms were recorded under nitrogen using TGA instrument TA Q50 V20.13 Build 39. Aluminium pans and a flow rate of 60 $cm^3$ $min^{-1}$ for the nitrogen gas were used for the experiments. The data was collected in the High Resolution Dynamic mode with a sensitivity of 1.0, a resolution of 4.0, and a temperature ramp of 10 °C $min^{-1}$ up to 550 °C. The data was evaluated using the T.A. Universal Analysis suite for Windows XP/Vista Version 4.5A.

**4. Dynamic Vapor Sorption (DVS)**

Dynamic water vapour sorption studies were performed on the sample using a dynamic vapour sorption system (Surface Measurement Systems, DVS Adventure) which gravimetrically measures the uptake and loss of vapour using air as a carrier gas. Pure water was used as the adsorbate for these measurements and temperature was maintained at 298 K by enclosing the system in a temperature-controlled incubator. Prior to measurement, the sample was in-situ activated at 100 °C, 0 RH. The mass of the sample was determined by comparison to an empty reference pan and recorded by a high resolution microbalance with a precision of 0.1 μg. Sorption isotherm was measured from 0 to 95% RH step-wise with a convergence equilibrium criterion dm/dt =0.05 %/min. The minimum and maximum equilibration times for each step were 10 and 360 min, respectively. The kinetics were measured between two points 0 and 30% RH with a convergence equilibrium criterion dm/dt =0.05 %/min. The recyclability test was done by performing 10 cycles, each cycle consisting of 15 min adsorption step (90% RH) and 30 min desorption step (0% RH).

## 5. Gas sorption measurements.

For gas sorption experiments, gases were used as received from BOC Gases Ireland: He (99.999%), $N_2$ (99.9995%), $CO_2$ (99.995%), $C_3H_4$ (97.0%), $C_3H_6$ (99.5%). Before sorption measurements, activation of **sql-NbOFFIVE-bpe-Cu-AB** and **sql-NbOFFIVE-bpe-Cu-AA** were achieved by degassing the air-dried samples on a SmartVacPrep™ using dynamic vacuum and heating for 12 h (the sample heated from RT to 333 K with a ramp rate of 1°C $min^{-1}$). About 100 mg of activated samples were used for the measurements on Micromeritics Tristar II 3030 or Micromeritics 3Flex surface area and pore size analyzer 3500. A Julabo temperature controller was used to maintain a constant temperature in the bath throughout the experiment. The bath temperatures of 273 and 298 K were precisely controlled with a Julabo ME (v.2) recirculating control system containing a mixture of ethylene glycol and water. The low temperatures at 77 K and 195 K were controlled by a 4 L Dewar filled with liquid $N_2$ and dry ice/acetone, respectively. At every interval of two independent isotherms recorded for any sorbent, samples were regenerated by degassing over 30 min under high vacuum at 333 K, before commencing the next sorption experiment.

## 6. Breakthrough experiments.

In typical breakthrough experiments, MeOH exchanged **sql-NbOFFIVE-bpe-Cu-AB** (~0.5 g) or MeOH exchanged **sql-NbOFFIVE-bpe-Cu-AA** (~0.35 g) was placed in quartz tubing (8 mm diameter; 8 mm x 6 mm x 400 mm) to form fixed beds. First, the adsorbent bed was purged under a 20 $cm^3$ $min^{-1}$ flow of He gas at 353 K for 6 hours prior to breakthrough experiment. Upon cooling to room temperature, the gas flow was switched to the desired $C_3H_4/C_3H_6$ gas mixture compositions (1:99), maintained at a total flow rate of 1.0 $cm^3$ $min^{-1}$. Then, 1:99 $C_3H_4/C_3H_6$ binary breakthrough experiments were conducted at 298 K. The outlet composition

was continuously monitored by a Shimadzu Nexis GC-2030 gas chromatograph until complete breakthrough was achieved.

The $C_3H_6$ productivity (q) is defined by the breakthrough amount of $C_3H_6$, which is calculated by integration of the breakthrough curves f(t) during a period from $\tau_1$ to $\tau_2$ where the $C_3H_6$ purity is higher than or equal to a threshold value p:

$$q = \frac{C_i(C_3H_6)}{C_i(C_3H_6) + C_i(C_3H_4)} \times \left(\int_{\tau_1}^{\tau_2} f(t)\,dt\right)$$

## 7. Single Crystal X-ray diffraction

Single crystal X-ray diffraction data were collected on a Bruker Quest diffractometer equipped with a I$\mu$S microfocus X-ray source Cu Kα, (λ = 1.54178 Å); Mo Kα, (λ = 0.71073 Å); Synchrotron (λ = 0.4859 Å), and CMOS detector. APEX3 or APEX4 was used for collecting, indexing, integrating and scaling the data.[1] An open-flow nitrogen attachment (Oxford Cryosystems) was used for low temperature measurements. Absorption corrections were performed by multi-scan method.[2] Space groups were determined using XPREP[3] as implemented in APEX3. All the scaled data were solved using intrinsic phasing method (XT)[4] and refined on $F^2$ using SHELXL[5] inbuilt in OLEX2 v1.5 (2020) program.[6] All non-hydrogen atoms present in the frameworks were refined anisotropically. Hydrogen atoms were located at idealized positions from the molecular geometry and refined isotropically with thermal parameters based on the equivalent displacement parameters of their carriers. Crystallographic data reported in this paper are summarized in Tables S4 and Table S5. These crystal structures have been deposited to the Cambridge Crystallographic Data Centre (CCDC 2154469-2154473, and 1973753).

## 8. Fitting of experimental data on pure component isotherms

The isotherm data for $C_3H_4$ in the flexible MOF at 298 K were fitted with the 2-site Langmuir-Freundlich model,[8] where we distinguish two distinct adsorption sites A and B:

$$q = q_{A,sat} \frac{b_A p^{\nu_A}}{1 + b_A p^{\nu_A}} + q_{B,sat} \frac{b_B p^{\nu_B}}{1 + b_B p^{\nu_B}} \tag{1}$$

The isotherm data for $C_3H_6$ in the flexible MOF at 298 K were fitted with the 3-site Langmuir-Freundlich model,[9, 10] where we distinguish three distinct adsorption sites A, B, and C:

$$q = q_{A,sat} \frac{b_A p^{\nu_A}}{1 + b_A p^{\nu_A}} + q_{B,sat} \frac{b_B p^{\nu_B}}{1 + b_B p^{\nu_B}} + q_{C,sat} \frac{b_C p^{\nu_C}}{1 + b_C p^{\nu_C}} \tag{2}$$

The unary isotherm fit parameters are provided in Tables S6 and S7.

**9. Adsorption Selectivity Calculations.**

The detailed methodology for calculating the amount of A and B adsorption from a mixture by ideal adsorbed solution theory (IAST) is described elsewhere.[11] The adsorption selectivity is finally defined as:

$$Selectivity = \frac{q_A / q_B}{p_A / p_B} \tag{3}$$

where $q_i$ (i = A or B) is the uptake quantity in the mixture and pi is the feeding partial pressure of component i.

**10. Breakthrough simulations**

The performance of industrial fixed bed adsorbers is dictated by a combination of adsorption selectivity and uptake capacity. Transient breakthrough simulations were carried out for 1/99 $C_3H_4/C_3H_6$ mixtures in the flexible MOF operating at a total pressure of 100 kPa, and temperatures of 298 K, using the methodology described in earlier publications.[12-16] In these simulations the intra-crystalline diffusional influences are considered to be of negligible importance. The breakthrough simulations are plotted as follows. The *y*-axis is the

dimensionless concentrations of each component at the exit of the fixed bed, normalized with respect to the inlet feed concentrations, $c / c_0$. The *x*-axis is

$$\frac{t}{m_{ads}} = \frac{(\text{time in min})}{(\text{g MOF packed in tube})} = \text{min g}^{-1}$$

**11. Separation factor / Separation selectivity calculations.**

The amount of adsorbed gas *i* ($q_i$) is calculated from the breakthrough curve as follows:

$$q_i = \frac{V_i T_0 - V_{dead} - \int_0^{t_0} V_e \Delta T}{m}$$

Here, $V_i$ is the influent flow rate of gas ($cm^3$ $min^{-1}$), $V_e$ is the effluent flow rate of gas ($cm^3$ $min^{-1}$), $V_{dead}$ is the dead volume of the system ($cm^3$), $T_0$ is the adsorption time (min) and *m* is the mass of the sorbent (g).[17]

On approximation, this simplifies to:

$$q_i = \frac{V_T \Delta T P_i}{m}$$

$V_T$ is the total flow rate of gas ($cm^3$ $min^{-1}$), $P_i$ is the partial pressure of gas *i* (bar) and $\Delta T$ is the time for initial breakthrough of gas *i* to occur (min).[18] The separation factor, also known as separation selectivity ($\alpha_{AC}$) for the breakthrough experiment *i.e.* breakthrough derived selectivity is determined as follows:

$$\alpha = \frac{q_{1\,y_2}}{q_{2\,y_1}}$$

$y_i$ is the partial pressure of gas *i* in the gas mixture. In the case where one gas component has negligible adsorption, the amount of gas adsorbed is treated as ≤ 1 $cm^3$ for calculations.

**12. Modeling Studies**

**Periodic Density Functional Theory (DFT) calculations** were performed using the projected augmented wave (PAW) formalism[33] as implemented in the Vienna Ab Initio Simulation Package (VASP 5.4.4),[34, 35] on 1x2x1 and 2x2x1 supercells for **sql-NbOFFIVE-bpe-Cu-AA-α,** and **sql-NbOFFIVE-bpe-Cu-AB-α**, respectively, employing the BEEF-vdW exchange-correlation functional.[36] Subsequently, the adsorption of $C_3H_4$ and $C_3H_6$ was studied in both supercells, and plausible binding sites were optimized at the Γ-point (using the experimental cell parameters) with the conjugate gradient algorithm with force and electronic convergence criteria of 0.01 eV/Å and $10^{-6}$ eV, a Gaussian smearing of 0.02 eV, an energy cut-off of 550 eV, and assuming a ferromagnetic coupling between of the unpaired electrons on each Cu atom (one unpaired electron per Cu). A partial Hessian vibrational analysis was performed by numerically displacing the atomic coordinates of the investigated adsorbates in x, y, and z-directions with ±0.01 Å to verify the binding sites as local minima on the potential energy surface. Furthermore, the adsorption enthalpies (at 298 K) and zero-point corrected adsorption energies and adsorption Gibbs free energies (at 298 K) were calculated with the post-processing toolkit TAMKIN.[37]

**Canonical Monte Carlo (CMC) simulations**[38] were performed to confirm the main binding site locations for the adsorbates $C_3H_4$ and $C_3H_6$ in the frameworks. CMC simulations were performed using RASPA[39, 40], and Materials Studio[41] at 298.15 K on 1x2x1 and 2x2x1 supercells of **sql-NbOFFIVE-bpe-Cu-AA-α** and **sql-NbOFFIVE-bpe-Cu-AB-α**, respectively, with each of the supercells containing 4 $Cu_2Nb_2O_2F_{10}(bpe)_4$ formula units. For the CMC simulations, the frameworks were considered rigid with atoms fixed at their DFT-optimized positions, and a fixed loading of eight sorbate molecules (per supercell) was considered, which corresponds with a loading of 1 adsorbate molecule per Cu and is about half of the maximum loading observed in experiments (Figure 2). In the canonical ensemble, the Metropolis sampling method

considered different moves, such as translation (corresponds to translation of the center-of-mass of the selected adsorbate molecule), rotation (rotating the selected adsorbate molecule), regrowth (removing a selected adsorbate molecule from the system and reintroduces it at a random position with random orientation), and conformer (collects sorbates multiple conformations), with relative probabilities of 1, 1, 0.1 and 1, respectively. Each CMC simulation included $2 \times 10^6$ loading steps, followed by $2 \times 10^6$ equilibration steps, and finally, $2 \times 10^6$ production steps to ensure reasonable ensemble averages. The output of the CMC simulations can be visualized as an isosurface (Table S11 and S12, Figures S31 and S32), encompassing the mass-middle points of all successfully inserted adsorbates during adsorbate insertion moves. The force field for the CMC simulations was parameterized as follows; the point charges for all framework atoms and adsorbates were determined via the extended charge equilibration (EQeq) method,[42] as implemented in the RASPA.[39, 40] The Lennard-Jones (LJ) pair coefficients were taken from the Universal Force Field (UFF).[43, 44] The applied Lennard-Jones pair coefficients for framework and adsorbates are given in Table S10. The point charges used for the adsorbates are given in Figure S30, while the point charges [e] for the framework atoms are supplied in Supplementary CIF-files (AA_type_opt_with_EQeq_point_charges.cif, AB_type_opt_with_EQeq_point_charges.cif). To compute the van der Waals potentials, the Lorentz-Berthelot mixing rules were used to calculate LJ interaction parameters between unlike atoms ($\varepsilon_{ij} = \sqrt{\varepsilon_i \varepsilon_j}$ and $\sigma_{ij} = \frac{1}{2}\left(\sigma_{ij} + \sigma_{ij}\right)$, where $\varepsilon$ is the depth of the potential well, $\sigma$ is the finite distance at which the inter-particle potential is zero).

# Physical and Electronic Parameters of Sorbent

**Table S1.** List of physical and electronic parameters for the $C_3H_4$, and $C_3H_6$.

| | 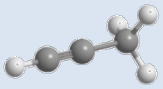 | 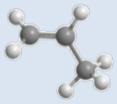 |
|---|---|---|
| Adsorbate molecules | $C_3H_4$ (propyne) | $C_3H_6$ (propylene) |
| Boiling point (K) | 250 | 225 |
| Dipole moment (x$10^{18}$ / e.s.u. cm) | 0.75 | 0.366 |
| Polarizability (x$10^{-25}$ $cm^3$) | 55.5 | 62.6 |
| Kinetic diameter (Å) | 4.2 | 4.7 |

# Summary of hybrid ultramicroporous materials (HUMs)

**Table S2.** Typical rigid bipyridine based ligands used to construct inorganic pillar based hybrid ultramicroporous materials (HUMs) with three dimensional networks.[a]

| Linker | Compound | Refcode | Year | Ref. |
|---|---|---|---|---|
| 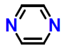 | **SIFSIX-3-Zn** | FUDQIF | 2013 | 19 |
| 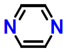 | **AlFFIVE-1-Ni** | DAXNOI | 2017 | 20 |
| 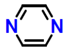 | **FeFIVE-1-Ni** | DAXPAW | 2017 | 20 |
| 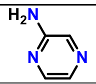 | **SIFSIX-17-Ni** | UQAVAM | 2021 | 21 |
| 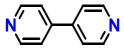 | **SIFSIX-1-Zn** | ZESFUY | 1995 | 22 |
| 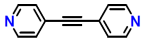 | **SIFSIX-2-Cu-i** | YEMTIV | 2013 | 19 |
| 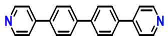 | **SIFSIX-5-Zn-i** | LIfWII | 2011 | 23 |

[a] i = interpenatrated

**Table S3.** Flexible bipyridine based ligands used to construct inorganic pillar based hybrid ultramicroporous materials (HUMs) with sql coordination networks.[b]

| Linker | Compound | Layer Arrangement | Solvent | Gas Separation | Year | Ref. |
|---|---|---|---|---|---|---|
| 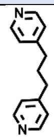 | $[Zn(SiF_6)(bpp)_2]_n$ | Coplanar | Methanol<br>Water | NP | 2006 | 24 |
| 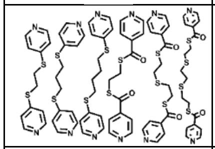 | $[Zn(SiF_6)L_2]_n$ | Coplanar | EtOH<br>$CHCl_3$ | NP | 2010 | 25 |
| 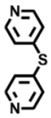 | $[Zn(dps)_2(SiF_6)]_n$<br>(**UTSA-300**) | Coplanar | Methanol<br>Methanol | $C_2H_2/CO_2$<br>$C_2H_2/C_2H_4$ | 2017 | 26 |
| | $[Cu(dps)_2(SiF_6)]$<br>(**NCU-100** or<br>**UTSA-300-Cu**) | Coplanar | Methanol<br>Water | $C_2H_2/C_2H_4$ | 2020 | 27 |
| | $[Cu(dps)_2(GeF_6)]_n$<br>(**GeFSIX-dps-Cu**)<br>$[Zn(dps)_2(GeF_6)]_n$<br>(**GeFSIX-dps-Zn**) | Coplanar | Methanol | $C_3H_4/C_3H_6$<br>$C_2H_2/C_2H_4$ | 2020 | 28 |
| 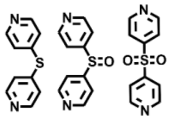 | $[Cu(L1)_2(NbOF_5)]_n$<br>(**ZUL-200**)<br>$[Cu(L2)_2(NbOF_5)]_n$<br>(**ZUL-210**)<br>$[Cu(dps)_2(NbOF_5)]_n$<br>(**ZUL-220**)<br>$[Cu(L1)_2(TiF_6)]_n$<br>(**ZUL-100**) | Coplanar | Methanol<br>DMF or<br>Water | $C_2H_2/C_2H_4$ | 2020 | 29 |
| 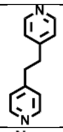 | $[Zn(SiF_6)(bpe)_2]_n$<br>(**sql-SISIX-bpe-Zn**) | Coplanar | Methanol<br>Methanol | $C_2H_2/CO_2$<br>$C_2H_2/C_2H_4$ | 2021 | 30 |
| 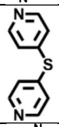 | $[Cu(dps)_2(SiF_6)]$<br>(**SIFSIX-dps-Cu** or<br>**NCU-100**) | Coplanar | Methanol<br>Water | $C_2H_2/CO_2$ | 2022 | 31 |
| 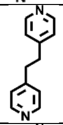 | $[Cu(NbOF_5)(bpe)_2]_n$<br>(**sql-NbOFFIVE-bpe-Cu-AA**) | **Coplanar** | **Methanol<br>Water** | **$C_3H_4/C_3H_6$** | **-** | **This work** |
| 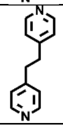 | $[Cu(NbOF_5)(bpe)_2]_n$<br>(**sql-NbOFFIVE-bpe-Cu-AB**) | **One layer rotated for $90^oC$** | **1,2-Dichlorobenzene<br>Water** | **$C_3H_4/C_3H_6$** | **-** | **This work** |

[b] Abbreviations: NP = not provided; bpp = 1,3-Bis(4-pyridyl)propane; L = 1,2-bis(pyridin-4-ylthio)ethane, or 1,3-bis(pyridin-4-ylthio)propane, or 1,4-bis(pyridin-4-ylthio)butane, or S,S'-(ethane-1,2-diyl) bis(pyridine-4-carbothioate), or S,S'-(thiobis(ethane-2,1-diyl)) bis(pyridine-4-carbothioate), or S,S'-((ethane-1,2-diylbis(sulfanediyl))bis(ethane-2,1-diyl)) bis(pyridine-4-

carbothioate); dps = 4,4'-dipyridylsulfide; L1 = 4,4'-dipyridylsulfone; L2 = 4,4'-dipyridylsulfoxide; bpe = 1,2-bis(4pyridyl)ethane.

## Tables of Crystal Data

**Table S4.** Crystallographic data and refinement parameters of **pcu-NbOFFIVE-bpe-Cu**, **sql-NbOFFIVE-bpe-Cu-AA-α** and **-β**.

| | **pcu-NbOFFIVE-bpe-Cu** | **sql-NbOFFIVE-bpe-Cu-AA-α** | **sql-NbOFFIVE-bpe-Cu-AA-β** |
|---|---|---|---|
| CCDC Number | 2154469 | 2154470 | 2154471 |
| Empirical formula | $C_{24}H_{24}CuF_5N_4NbO$ | $C_{26}H_{32}CuF_5N_4NbO_3$ | $C_{24}H_{24}CuF_5N_4NbO$ |
| Formula weight | 635.92 | 700.00 | 635.92 |
| Temperature/K | 120 | 100.00 | 333.00(10) |
| Crystal system | tetragonal | monoclinic | monoclinic |
| Space group | *P*4/*n* | *C*2/*c* | *I*2/*m* |
| a/Å | 18.6068(3) | 20.1537(5) | 8.5344(10) |
| b/Å | 18.6068(3) | 8.5345(2) | 18.513(2) |
| c/Å | 8.3522(2) | 19.4208(4) | 9.5971(11) |
| α/° | 90 | 90 | 90 |
| β/° | 90 | 117.2220(10) | 96.835(11) |
| γ/° | 90 | 90 | 90 |
| Volume/Å$^3$ | 2891.64(12) | 2970.43(12) | 1505.5(3) |
| Z | 2 | 4 | 2 |
| $\rho_{calc}$g/cm$^3$ | 0.730 | 1.565 | 1.403 |
| μ/mm$^{-1}$ | 0.593 | 4.608 | 1.138 |
| Radiation | Mo Kα | Cu Kα | Mo Kα |
| Reflections collected | 43292 | 12499 | 12846 |
| Independent reflections | 3531 | 2287 | 1379 |
| Goodness-of-fit on F$^2$ | 1.079 | 1.079 | 1.046 |
| $R_1$[I>2σ(I)][c] | 0.0825 | 0.0554 | 0.0792 |
| $wR_2$[I>2σ(I)][d] | 0.2765 | 0.1627 | 0.2445 |

[c] $R_1 = \Sigma||F_o| - |F_c||/\Sigma|F_o|$.    [d] $wR_2 = \{\Sigma[w(F_o^2 - F_c^2)^2]/\Sigma[w(F_o^2)^2]\}^{1/2}$

**Table S5.** Crystallographic data and refinement parameters of **sql-NbOFFIVE-bpe-AB-α** phase and **-β$_1$** phase.

| | **sql-NbOFFIVE-bpe-AB-α** | **sql-NbOFFIVE-bpe-AB-β$_1$** | **sql-NbOFFIVE-bpe-AB-β$_1$ (Synchrotron)** |
|---|---|---|---|
| CCDC Number | 2154472 | 2154473 | 1973753 |
| Empirical formula | $C_{24}H_{24}CuF_5N_4NbO$ | $C_{24}H_{24}CuF_5N_4NbO$ | $C_{24}H_{26}CuF_5N_4NbO_2$ |
| Formula weight | 635.92 | 635.92 | 653.94 |
| Temperature/K | 295.0(2) | 293(2) | 293(2) |
| Crystal system | tetragonal | tetragonal | tetragonal |
| Space group | $P4_2/mmc$ | $P4_2/mnm$ | P4$_2$/mnm |
| a/Å | 9.0177(7) | 12.5384(7) | 12.40990(10) |
| b/Å | 9.0177(7) | 12.5384(7) | 12.40990(10) |
| c/Å | 18.9558(15) | 18.7936(12) | 18.8877(3) |
| α/° | 90 | 90 | 90 |
| β/° | 90 | 90 | 90 |
| γ/° | 90 | 90 | 90 |
| Volume/Å$^3$ | 1541.5(3) | 2954.6(4) | 2908.81(7) |
| Z | 2 | 4 | 4 |
| $\rho_{calc}$g/cm$^3$ | 1.37 | 1.43 | 1.493 |
| μ/mm$^{-1}$ | 4.337 | 1.16 | 1.141 |
| Radiation | Cu Kα | Mo Kα | Synchrotron |
| Reflections collected | 3988 | 30190 | 81311 |
| Independent reflections | 894 | 1502 | 5243 |
| Goodness-of-fit on F2 | 1.081 | 1.098 | 0.992 |
| $R_1$[I>2σ(I)][c] | 0.0856 | 0.0998 | 0.0624 |
| $wR_2$[I>2σ(I)][d] | 0.2632 | 0.2436 | 0.2372 |

[c] $R_1 = \Sigma||F_o| - |F_c||/\Sigma|F_o|$.    [d] $wR_2 = \{\Sigma[w(F_o^2 - F_c^2)^2]/\Sigma[w(F_o^2)^2]\}^{1/2}$.

## Optical Image

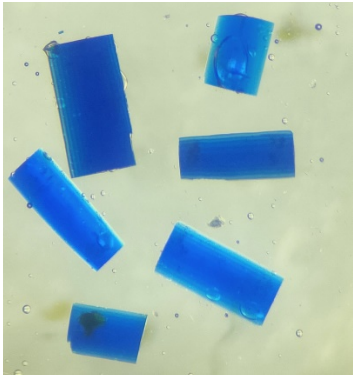

**Figure S1.** Optical image of as-synthesized **sql-NbOFFIVE-bpe-Cu-AA-α.**

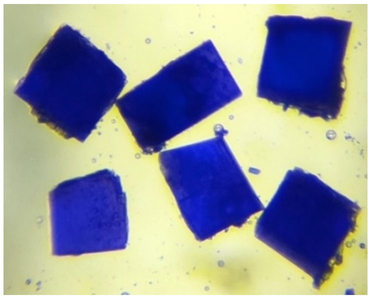

**Figure S2.** Optical image of as-synthesized **sql-NbOFFIVE-bpe-Cu-AB-α**.

## Crystal Structures

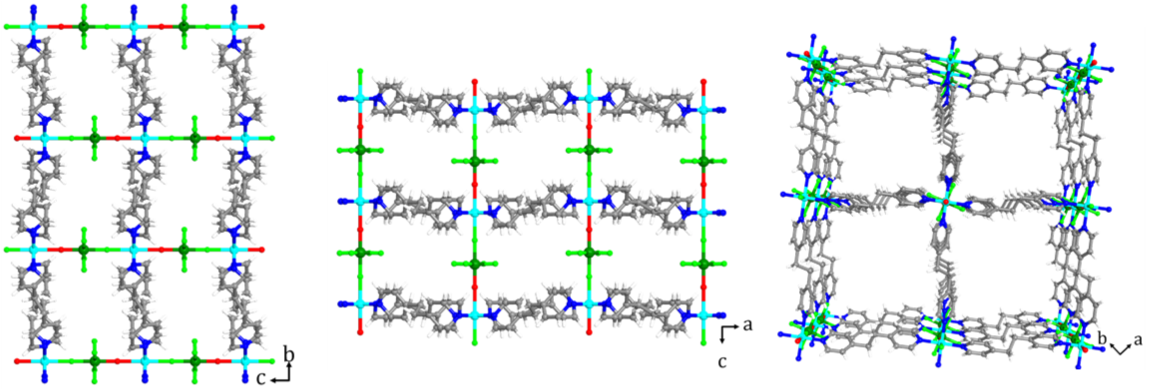


**Figure S3.** Structural representations of **pcu-NbOFFIVE-bpe-Cu**, a 3D non-interpenetrated network, across different planes.

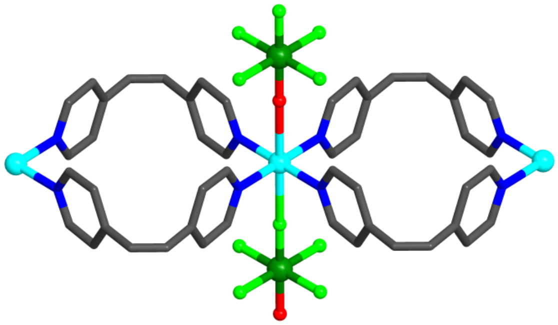

**Figure S4**. Coordination environment of $Cu^{2+}$ cation in the structures of **sql-NbOFFIVE-bpe-Cu-AA-α** and **sql-NbOFFIVE-bpe-Cu-AB-α**.

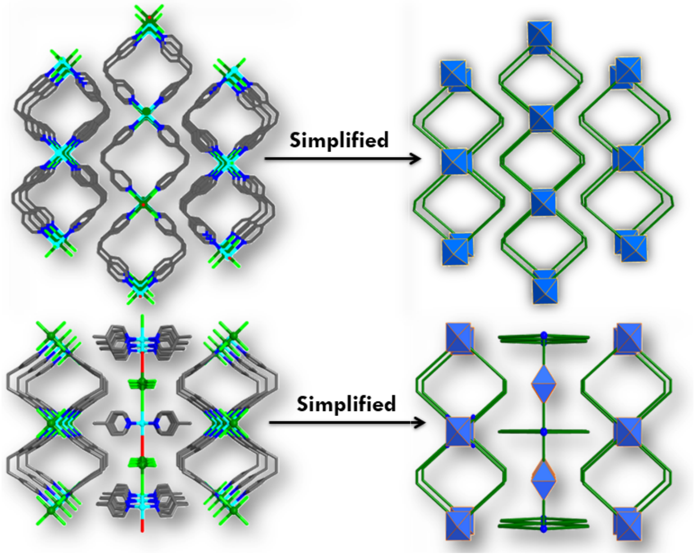


**Figure S5.** Simplified structures of **sql-NbOFFIVE-bpe-Cu-AA-α** (above array) and **sql-NbOFFIVE-bpe-Cu-AB-α** (below array).

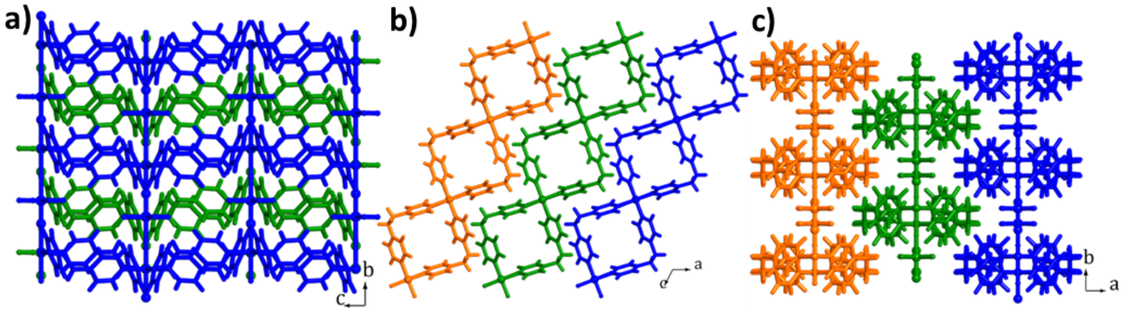


**Figure S6.** Packing structures for **sql-NbOFFIVE-bpe-Cu-AA-α** along with the [100], [010], and [001] direction, respectively.

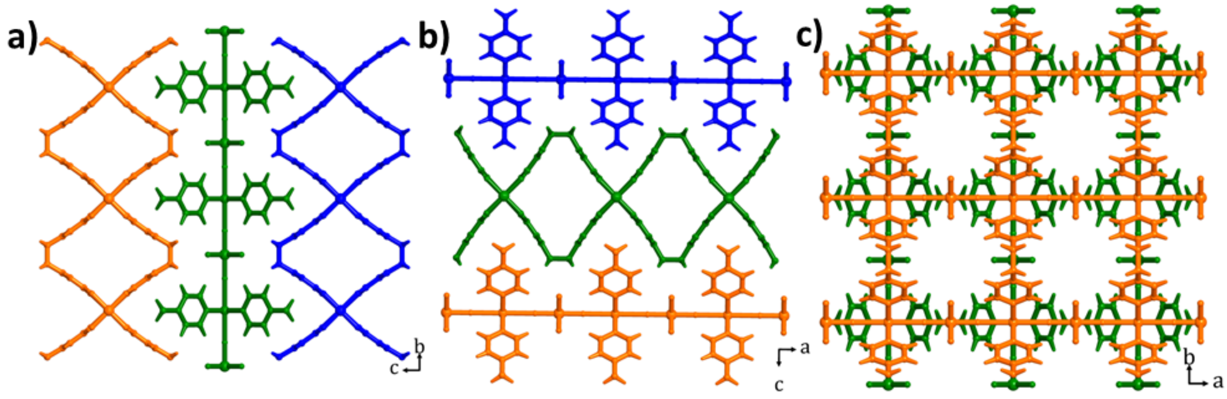


**Figure S7.** Packing structures for **sql-NbOFFIVE-bpe-Cu-AB-α** along with the [100], [010], and [001] direction, respectively.

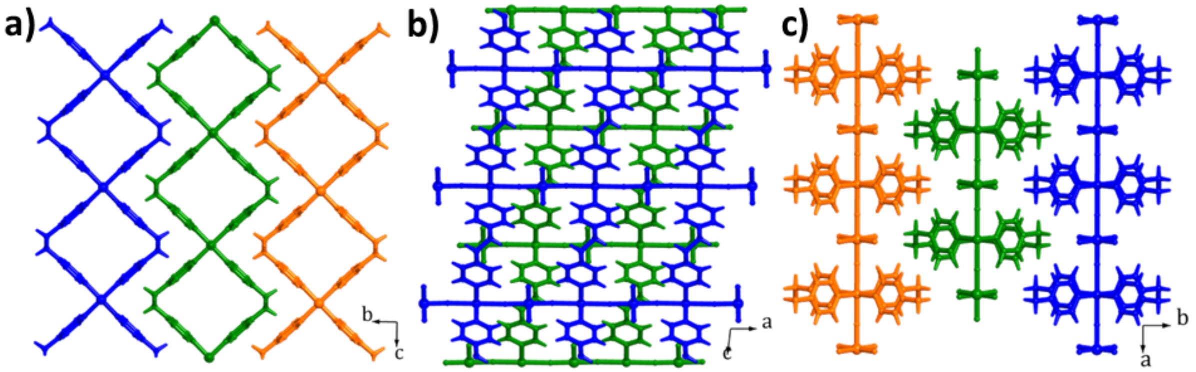


**Figure S8.** Packing structures of **sql-NbOFFIVE-bpe-Cu-AA-β** along with the [100], [010], and [001] direction, respectively.

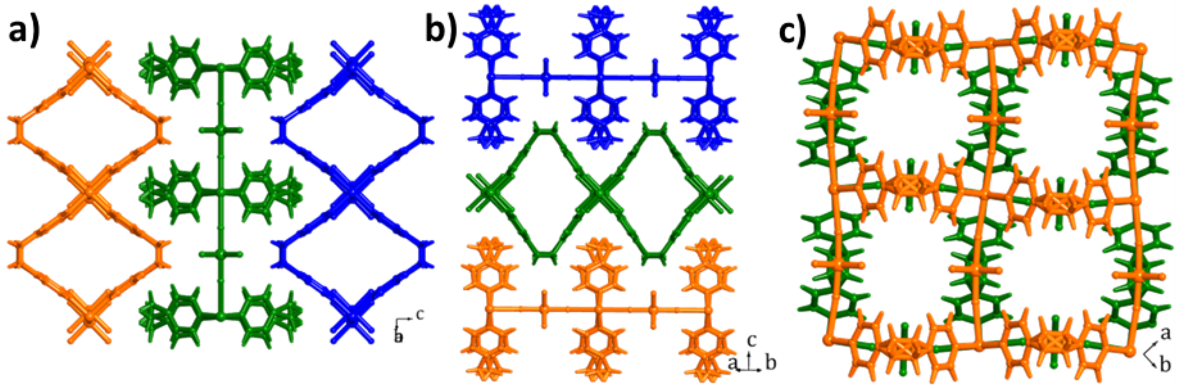


**Figure S9.** Packing structures of **sql-NbOFFIVE-bpe-Cu-AB-β$_1$** along with the [1$\bar{1}$0], [110], and [001] direction, respectively.

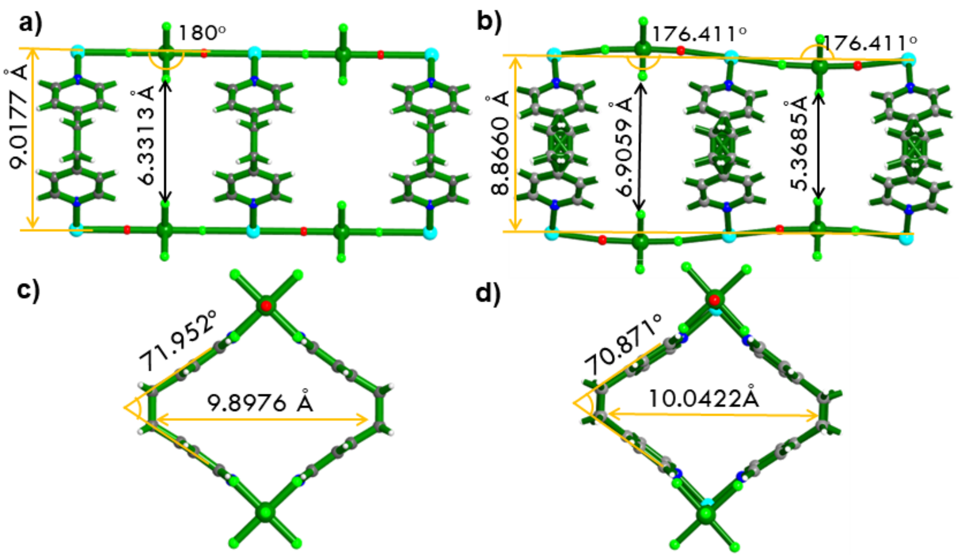


**Figure S10.** Comparison of different phases for **sql-NbOFFIVE-bpe-Cu-AB-α** (a, c) and **sql-NbOFFIVE-bpe-AB-β$_1$** (b, d) (Van der Waals radius is included for the distance measurements).

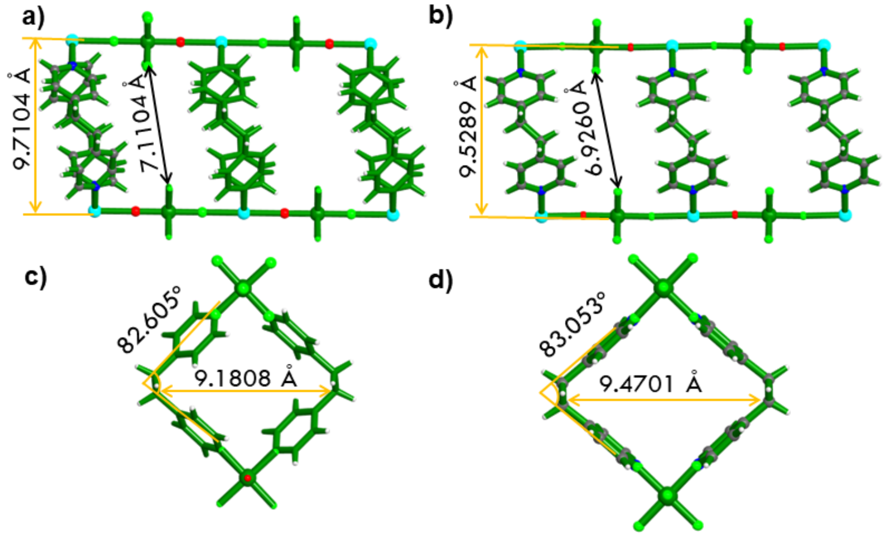


**Figure S11.** Comparison of Packing modes for **sql-NbOFFIVE-bpe-Cu-AA-α** (a, c) and **sql-NbOFFIVE-bpe-Cu-AA-β** (b, d) (Van der Waals radius is included for the distance measurements).

## PXRD Patterns

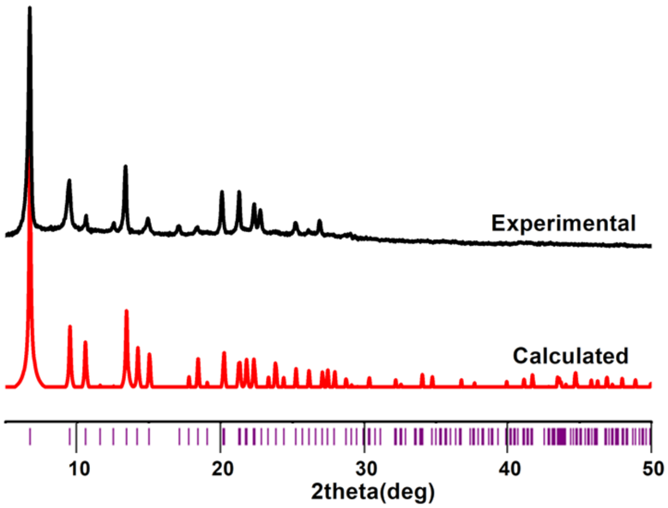


**Figure S12.** PXRD patterns of as-synthesized sample **pcu-NbOFFIVE-bpe-Cu**, along with the calculated XRD pattern from the single-crystal X-ray structure.

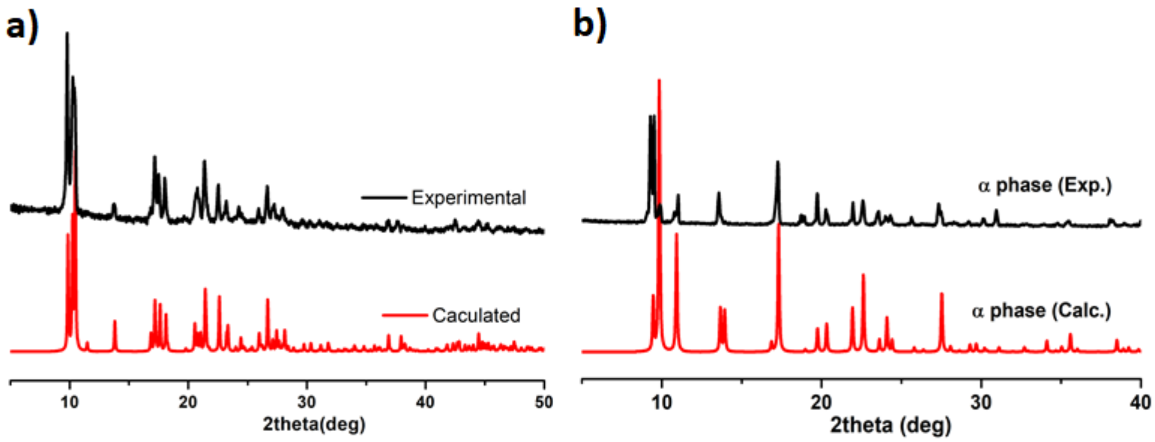


**Figure S13**. PXRD patterns of as-synthesized sample **sql-NbOFFIVE-bpe-Cu-AA-α** (a) and **sql-NbOFFIVE-bpe-Cu-AB-α** (b), along with the simulated XRD pattern from the single-crystal X-ray structure.

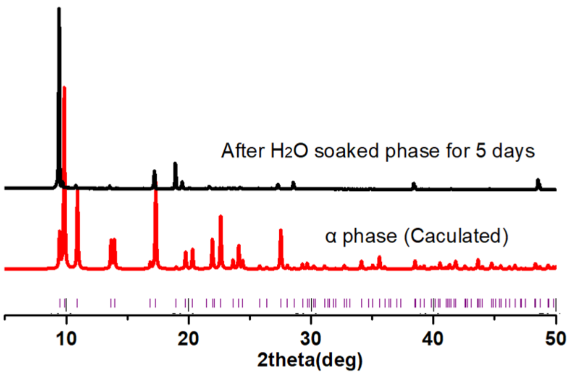


**Figure S14.** Comparison of experimental and calculated PXRD patterns of **sql-NbOFFIVE-bpe-Cu-AB-α** and water-soaked phase.

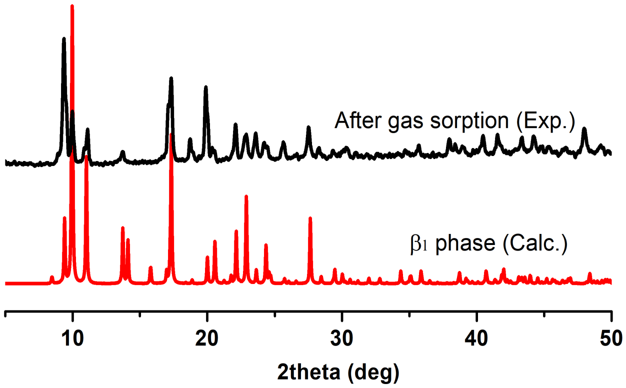


**Figure S15.** Comparison of PXRD patterns for **sql-NbOFFIVE-bpe-Cu-AB** after gas sorption and calculated $\boldsymbol{\beta_1}$ phase.

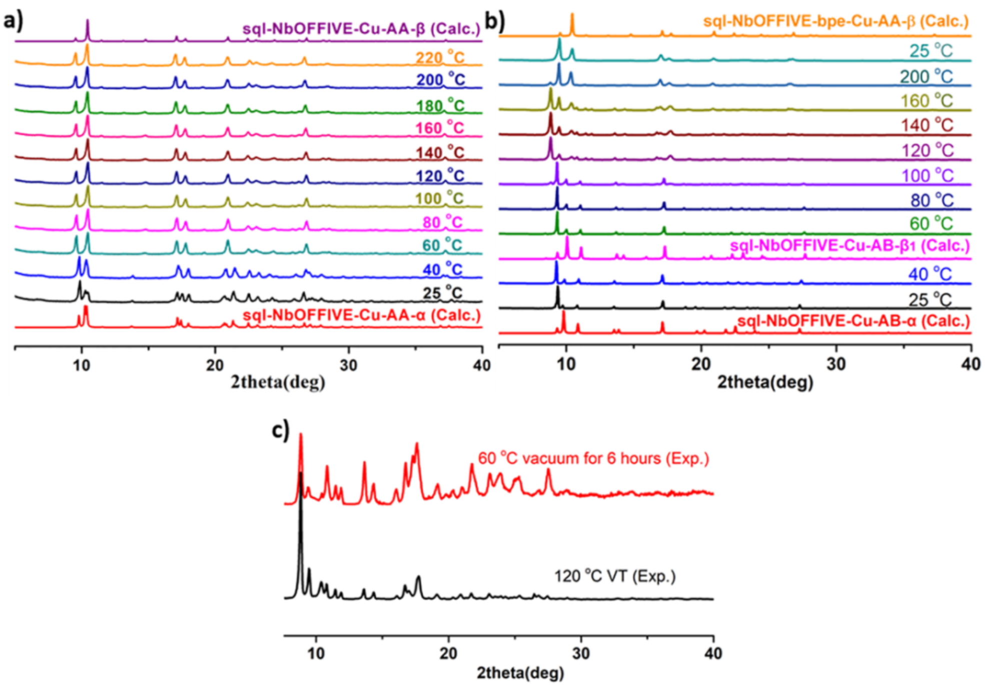


**Figure S16.** Variable temperature PXRD patterns for MeOH exchanged **sql-NbOFFIVE-bpe-Cu-AA** (a), **sql-NbOFFIVE-bpe-Cu-AB** (b), and comparison of PXRD patterns for MeOH exchanged **sql-NbOFFIVE-bpe-Cu-AB** after heating the sample at 60 °C under vacuum for 6 hours with VT-PXRD pattern at 120 °C (c).

# TGA Curves

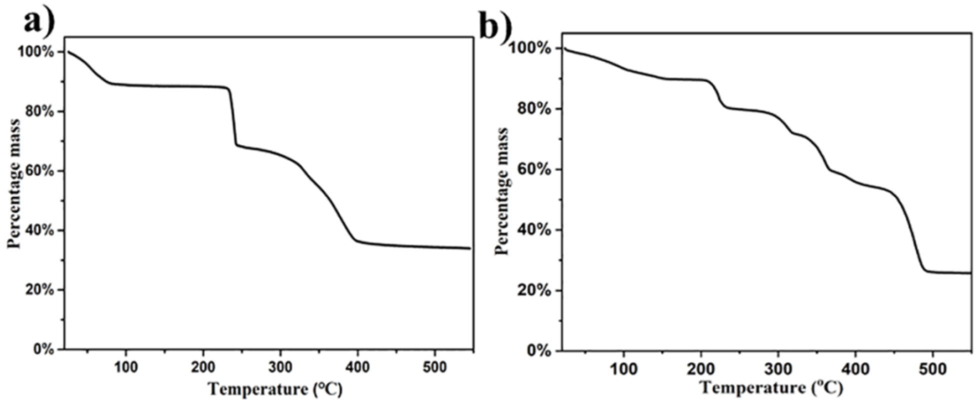


**Figure S17.** Thermogravimetric analysis profile of the as-synthesized sample **sql-NbOFFIVE-bpe-Cu-AA-α** (a) and **sql-NbOFFIVE-bpe-Cu-AB-α** (b).

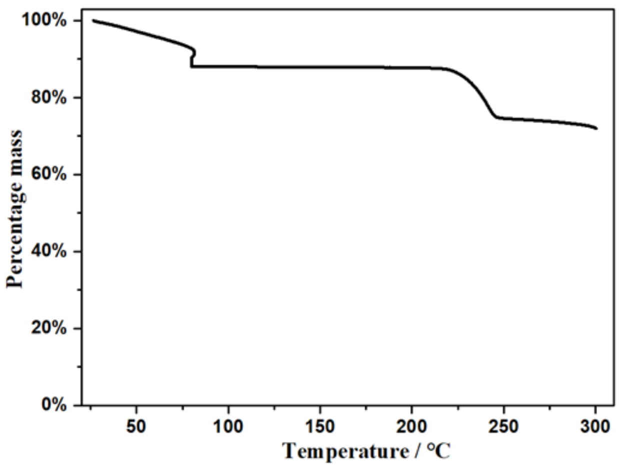


Procedure:
Step 1: Ramp 10.00 °C/min to 80.00 °C
Step 2: Isothermal for 120.00 min
Step 3: Mark end of cycle 1
Step 4: Ramp 10.00 °C/min to 140.00 °C
Step 5: Isothermal for 30.00 min
Step 6: Mark end of cycle 2
Step 7: Ramp 20.00 °C/min to 300.00 °C
Step 8: Isothermal for 5.00 min
Step 9: Mark end of cycle 3
Step 10: Ramp 20.00 °C/min to 25.00 °C
Step 11: Mark end of cycle 4

**Figure S18.** Thermogravimetric analysis profile of MeOH exchanged sample **sql-NbOFFIVE-bpe-Cu-AB-α** with different procrdure.

## Sorption Studies

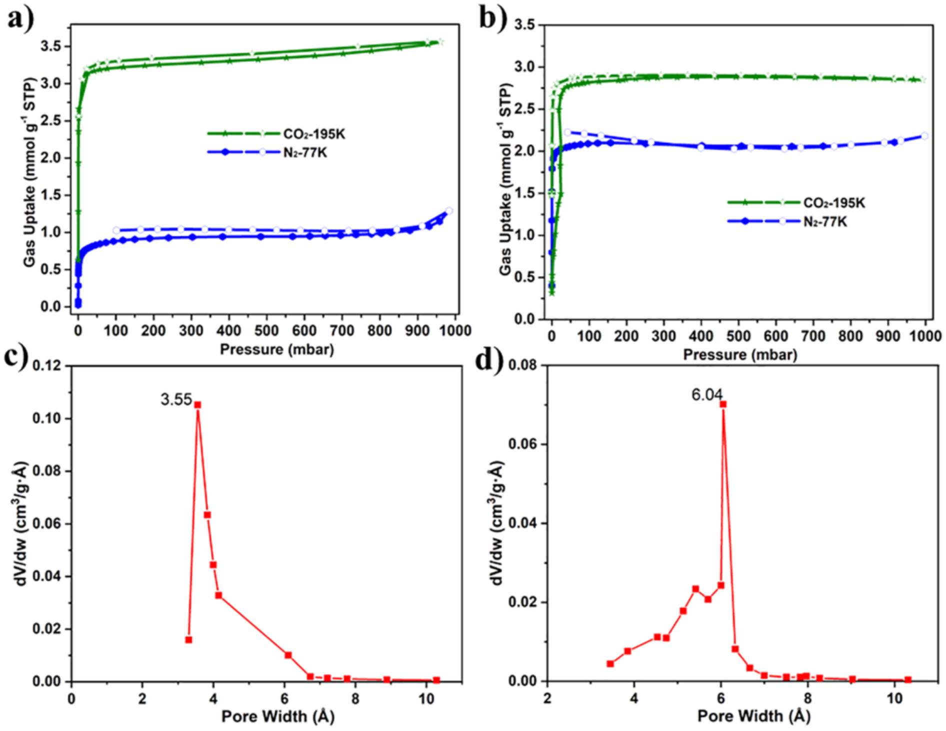


**Figure S19**. Low-temperature $CO_2$ and $N_2$ sorption isotherms of **sql-NbOFFIVE-bpe-Cu-AA** (a) and **sql-NbOFFIVE-bpe-Cu-AB** (b); Horvath-Kawazoe pore size distribution of **sql-NbOFFIVE-bpe-Cu-AA** (c) and **sql-NbOFFIVE-bpe-Cu-AB** (d) derived from 195 K $CO_2$.

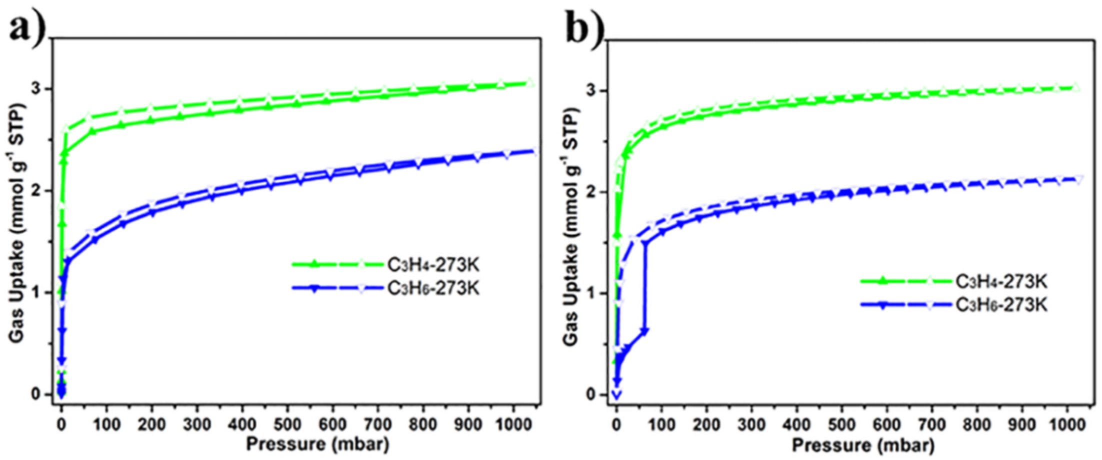


**Figure S20.** $C_3H_4$ and $C_3H_6$ adsorption isotherms of **sql-NbOFFIVE-bpe-Cu-AA** (a) and **sql-NbOFFIVE-bpe-Cu-AB** (b) at 273 K.

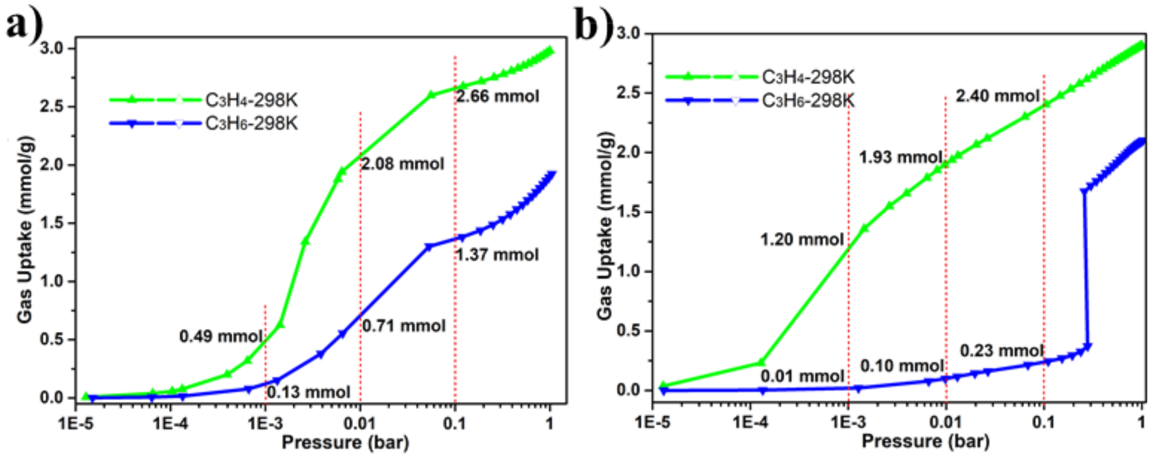


**Figure S21**. Log scale plot of single-component ($C_3H_4$ and $C_3H_6$) gas adsorption isotherms of **sql-NbOFFIVE-bpe-Cu-AA** (a) and **sql-NbOFFIVE-bpe-Cu-AB** (b) at 298 K (0-1 bar).

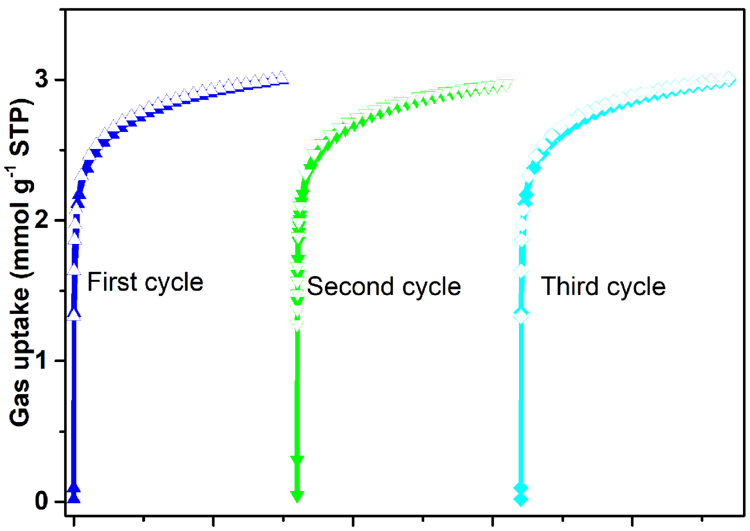


**Figure S22**. Three cycles of $C_3H_4$ adsorption and desorption on **sql-NbOFFIVE-bpe-Cu-AB** at 298 K (0-1 bar).

# Fitting Isotherms

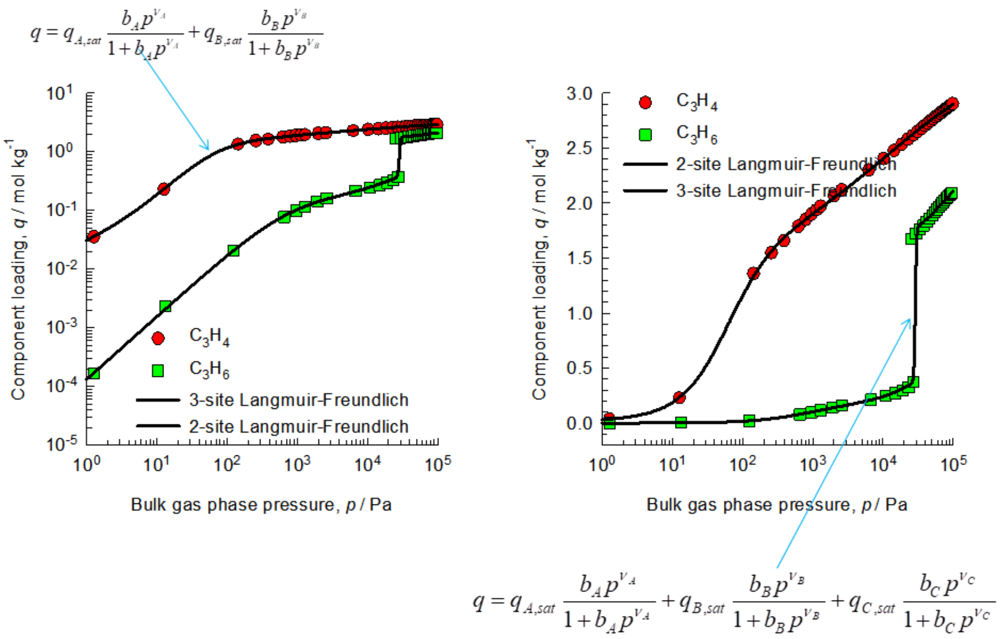


**Figure S23**. Fitting unary isotherms of $C_3H_4$ and $C_3H_6$ for **sql-NbOFFIVE-bpe-Cu-AB** at 298 K.

## IAST selectivity

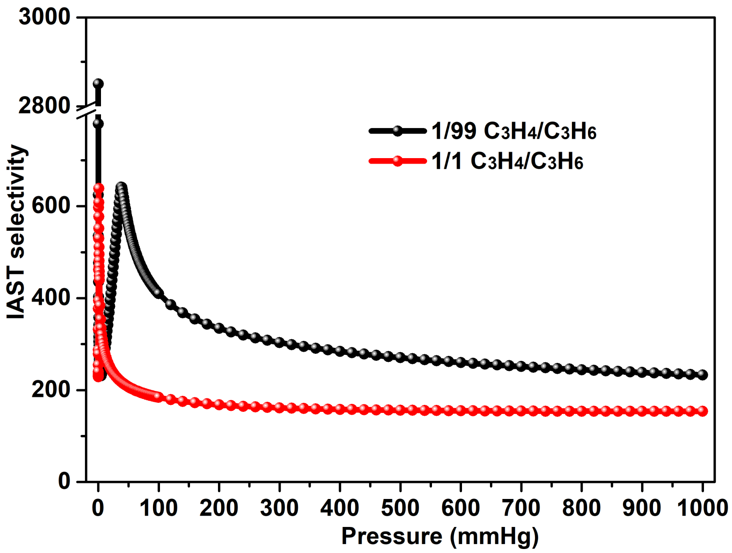


**Figure S24**. IAST selectivity of **sql-NbOFFIVE-bpe-Cu-AB** calculated for $C_3H_4/C_3H_6$ gas mixtures for 1:1 and 1:99 ratios at 298 K.

## Fitting Related Parameter

**Table S6**. 2-site Langmuir-Freundlich parameter fits for $C_3H_4$.

| | |
|---|---|
| $q_{A,sat}$ / mol kg$^{-1}$ | 1.46 |
| $q_{B,sat}$ / mol kg$^{-1}$ | 2 |
| $b_A$ / Pa$^{-\nu_A}$ | 3.691E-03 |
| $b_B$ / Pa$^{-\nu_B}$ | 1.292E-02 |
| $\nu_A$ / dimensionless | 1.36 |
| $\nu_B$ / dimensionless | 0.46 |

**Table S7**. 3-site Langmuir-Freundlich parameter fits for $C_3H_6$.

| | |
|---|---|
| $q_{A,sat}$ / mol kg$^{-1}$ | 1.39 |
| $q_{B,sat}$ / mol kg$^{-1}$ | 0.18 |

| | |
|---|---|
| $q_{C,sat}$ / mol kg$^{-1}$ | 1.2 |
| $b_A$ / Pa$^{-\nu_A}$ | 1.153E-245 |
| $b_B$ / Pa$^{-\nu_B}$ | 7.128E-04 |
| $b_C$ / Pa$^{-\nu_C}$ | 2.584E-06 |
| $\nu_A$ / dimensionless | 54.9 |
| $\nu_B$ / dimensionless | 1.072 |
| $\nu_C$ / dimensionless | 1.1 |

## Experimental Breakthrough Related Data

**Table S8**. The data related to breakthrough measurement for **sql-NbOFFIVE-bpe-Cu-AB** at 1.0 bar and 298 K.

| | **sql-NbOFFIVE-bpe-Cu-AB** |
|---|---|
| $C_3H_4$ inlet flow (cc min$^{-1}$) | 0.01 |
| $C_3H_6$ inlet flow (cc min$^{-1}$) | 0.99 |
| $C_3H_4$ uptake (mmol g$^{-1}$) | 1.20 |
| $C_3H_6$ uptake (mmol g$^{-1}$) | 0.44 |
| $C_3H_6$ productivity (mmol g$^{-1}$) | 118 |
| $\alpha_{AC}$ | 270 |
| Minimum effluent $C_3H_6$ purity | 99.99% |

## Gravimetric Kinetics

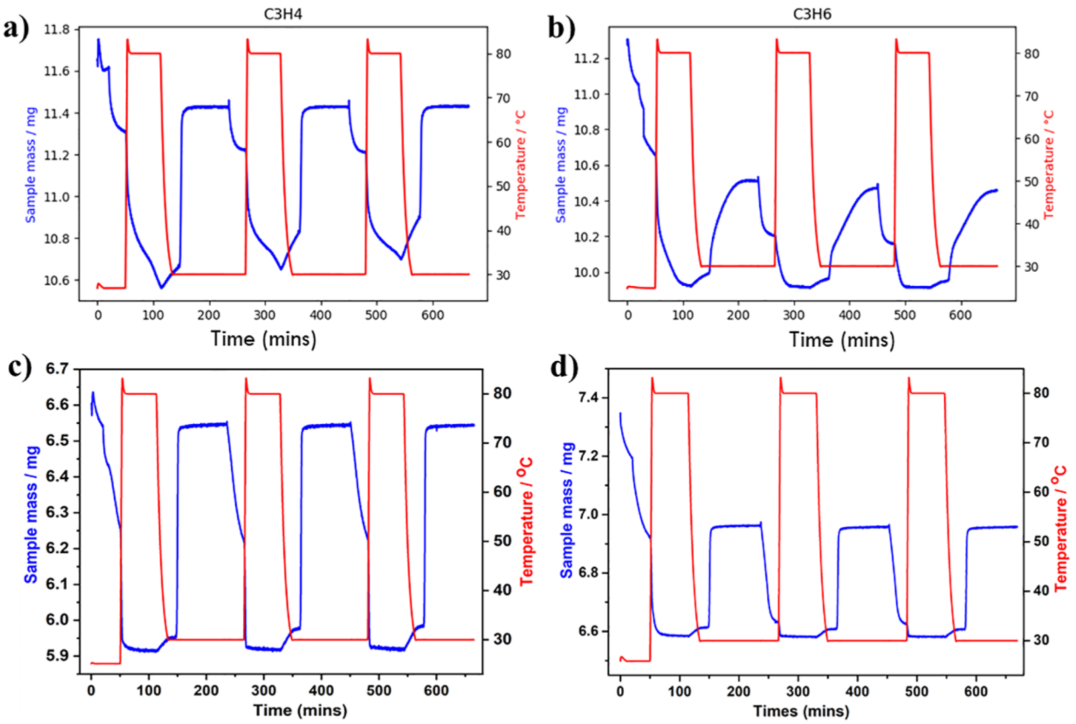


**Figure S25**. $C_3H_4$ and $C_3H_6$ adsorption kinetics plot at different temperatures and 1.0 bar for **sql-NbOFFIVE-bpe-Cu-AB** (a, b) and **sql-NbOFFIVE-bpe-Cu-AA** (c, d).

Gravimetric uptakes were recorded under pure $C_3H_4$ and $C_3H_6$ flows using TGA instrument TA Q50 V20.13 with sample mass 11.6540 mg and 11.3050 mg for **sql-NbOFFIVE-bpe-Cu-AB**, as well as 6.6080 mg and 7.3480 mg for **sql-NbOFFIVE-bpe-Cu-AA**, respectively. $C_3H_4$ and $C_3H_6$ flow rates of 10 $cm^3$/min were used in these experiments, and gas flows were controlled by pre-callibrated Bronkhorst Mass Flow Controllers. Desorption at 353 K was performed under $N_2$ flow of 60 $cm^3$ /min. The data was evaluated using the T.A. Universal Analysis suite for Windows. The procedure we used is listed as follows.

| Step | Action | Step | Action |
|---|---|---|---|
| 1 | Select gas 2 | 23 | Isothermal for 30.00 min |
| 2 | Mass flow 60.00 mL/min | 24 | Mark end of cycle 3 |

| | | | |
|---|---|---|---|
| 3 | Isothermal for 20.00 min | 25 | Select gas 2 |
| 4 | Mark end of cycle 0 | 26 | Mass flow 10.00 mL/min |
| 5 | Select gas 1 | 27 | Isothermal for 90.00 min |
| 6 | Mass flow 60.00 mL/min | 28 | Mark end of cycle 4 |
| 7 | Isothermal for 30.00 min | 29 | Select gas 1 |
| 8 | Ramp 20.00 °C/min to 80.00 °C | 30 | Mass flow 60.00 mL/min |
| 9 | Isothermal for 60.00 min | 31 | Isothermal for 30.00 min |
| 10 | Ramp 20.00 °C/min to 30.00 °C | 32 | Ramp 20.00 °C/min to 80.00 °C |
| 11 | Isothermal for 30.00 min | 33 | Isothermal for 60.00 min |
| 12 | Mark end of cycle 1 | 34 | Ramp 20.00 °C/min to 30.00 °C |
| 13 | Select gas 2 | 35 | Isothermal for 30.00 min |
| 14 | Mass flow 10.00 mL/min | 36 | Mark end of cycle 5 |
| 15 | Isothermal for 90.00 min | 37 | Select gas 2 |
| 16 | Mark end of cycle 2 | 38 | Mass flow 10.00 mL/min |
| 17 | Select gas 1 | 39 | Isothermal for 90.00 min |
| 18 | Mass flow 60.00 mL/min | 40 | Mark end of cycle 6 |
| 19 | Isothermal for 30.00 min | 41 | Select gas 1 |
| 20 | Ramp 20.00 °C/min to 80.00 °C | 42 | Mass flow 10.00 mL/min |
| 21 | Isothermal for 60.00 min | 43 | Mark end of cycle 8 |
| 22 | Ramp 20.00 °C/min to 30.00 °C | | |

Note: gas 2 is target gas ($C_3H_4$ or $C_3H_6$); gas 1 is $N_2$.

## Water Sorption

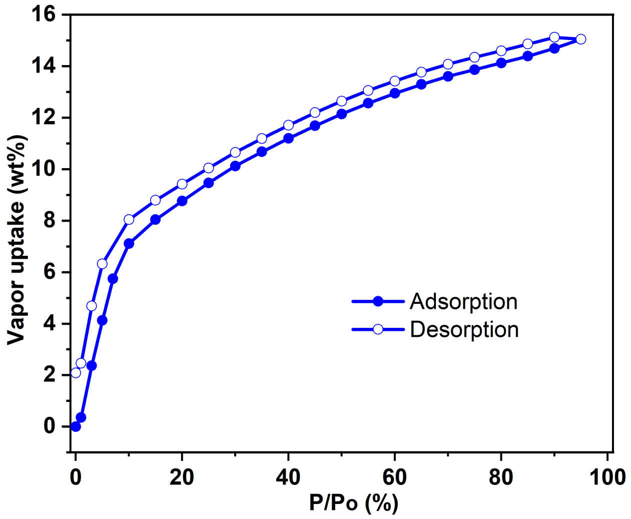


**Figure S26**. Water vapor sorption isotherm of **sql-NbOFFIVE-bpe-Cu-AB** at 298 K.

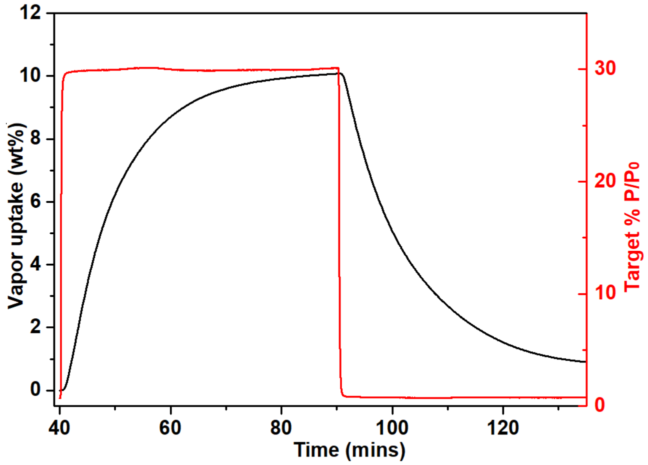


**Figure S27**. Vapor adsorption kinetics plot of **sql-NbOFFIVE-bpe-Cu-AB** at 298 K.

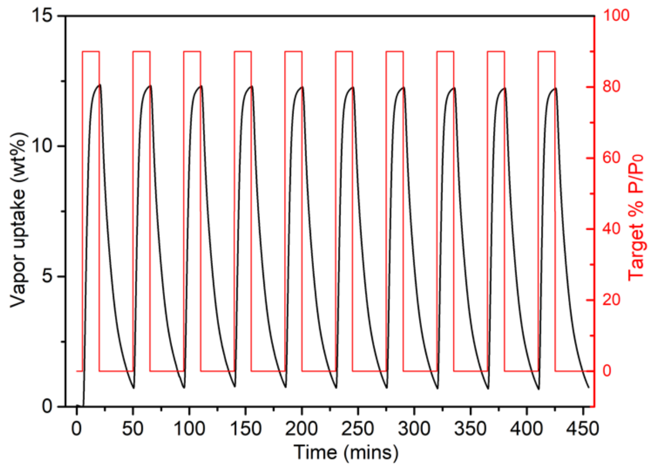


**Figure S28**. Adsorption-desorption cycles for **sql-NbOFFIVE-bpe-Cu-AB** at 298 K (adsorption at 90% RH and desorption at 0 RH).

# Experimental Setup of DCB

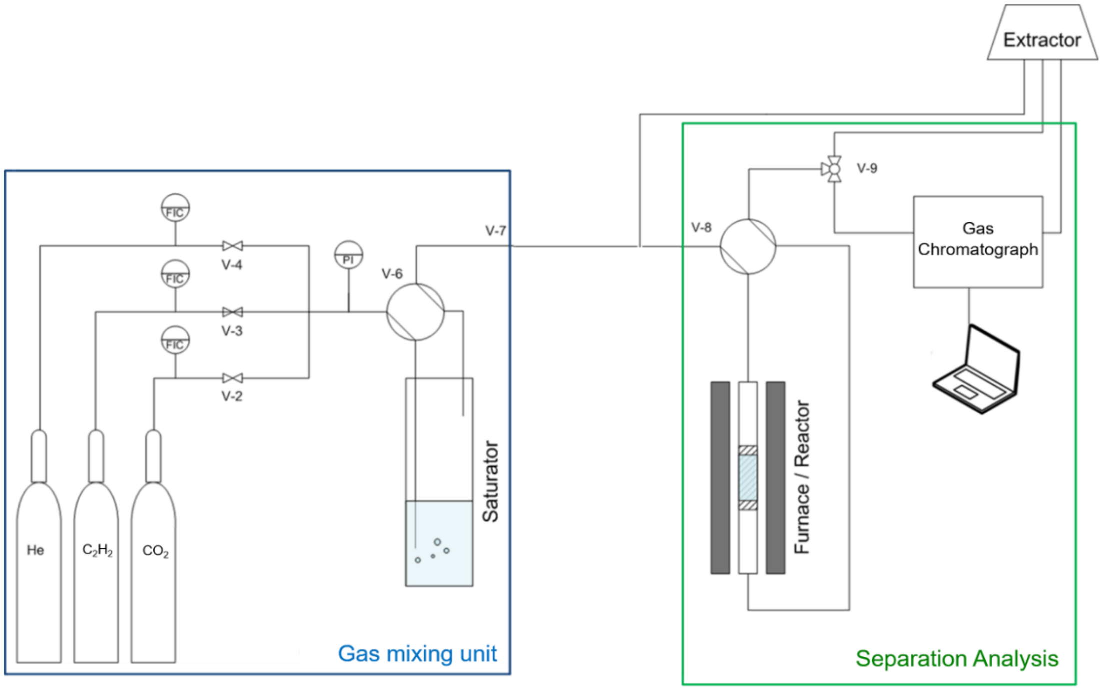


**Figure S29**. Schematic of dynamic gas breakthrough separation experimental setup, including gas mixing unit, gravimetric gas uptake analyser and gas separation analyser.[32]

## Modeling Studies

**Table S9.** Details on the binding sites (two possible orientations of the adsorbates), obtained from DFT calculations.

| Adsorbent | Adsorbate | File Name | Adsorption enthalpy (kJ $mol^{-1}$) | Adsorption Enthalpy | Distance (Å) |
|---|---|---|---|---|---|
| sql-NbOFFIVE-bpe-Cu-AA | $C_3H_4$ | sql-NbOFFIVE-bpe-Cu-AA-$C_3H_4$ binding site | -62.7 | C-H···F | 2.49, 2.60, 2.88, 2.61 |
| | | | | C-H(Ligand)···π($C_3H_4$) | 2.98 |
| | $C_3H_6$ | sql-NbOFFIVE-bpe-Cu-AA-$C_3H_6$ binding site | -65.2 | C-H···F | 2.50, 2.43, 2.58, 2.76 |
| | | | | C-H(Ligand)···π($C_3H_6$) | 3.09 |
| sql-NbOFFIVE-bpe-Cu-AB | $C_3H_4$ | sql-NbOFFIVE-bpe-Cu-AB-$C_3H_4$ binding site | -69.0 | C-H···F | 2.35, 2.36 |
| | | | | C-H(Ligand)···π($C_3H_4$) | 2.77, 3.04, 2.85, 3.10, 3.09 (with another layer) |
| | $C_3H_6$ | sql-NbOFFIVE-bpe-Cu-AB-$C_3H_6$ binding site | -53.0 | C-H···F | 2.64, 2.83, 2.91, 2.79, 2.43, 2.64, 2.93, 2.85 |
| | | | | C-H(Ligand)···π($C_3H_6$) | No |

**Table S10.** Lennard-Jones (LJ) interaction parameters representing framework atoms. The interaction with the sorbate molecules was calculated using Lorentz-Berthelot mixing rules.

| Atom | $\varepsilon/k_B$ [K] | σ [Å] |
|---|---|---|
| Nb | 29.68992 | 2.819694 |
| Cu | 2.516095 | 3.113691 |
| F | 25.16095 | 2.996983 |

| | | |
|---|---|---|
| O | 30.19314 | 3.118146 |
| N | 34.72211 | 3.260689 |
| C | 52.83799 | 3.430851 |
| H | 22.14164 | 2.571134 |
| $C_3H_4$ and $C_3H_6$ | | |
| C | 52.83799 | 3.430851 |
| H | 22.14164 | 2.571134 |

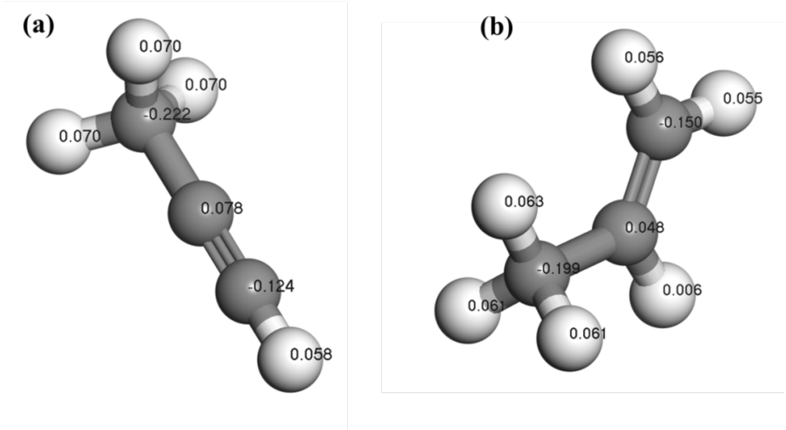


**Figure S30.** Atomic structures and point charges [e] of (a) $C_3H_4$ and (b) $C_3H_6$.

**Table S11.** Visualization from different view angles of $C_3H_4$ and $C_3H_6$ binding site isosurfaces from CMC simulations with 8 adsorbates in a 1x2x1 **sql-NbOFFIVE-bpe-Cu-AA-α** supercell.

| | **sql-NbOFFIVE-bpe-Cu-AA_$C_3H_4$** | **sql-NbOFFIVE-bpe-Cu-AA_$C_3H_6$** |
|---|---|---|
| **View 1** | 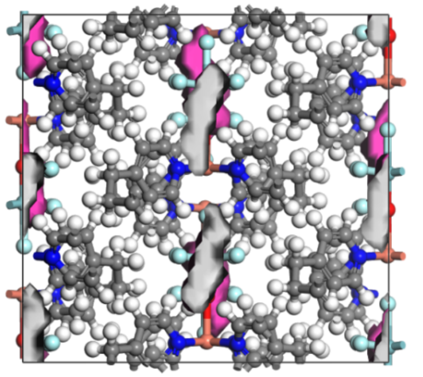 | 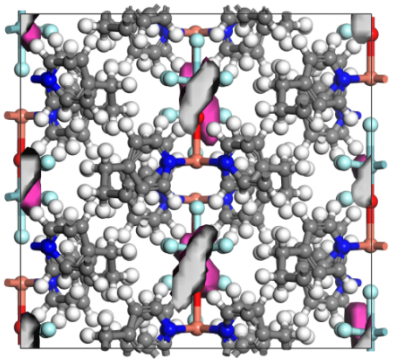 |
| **View 2** | 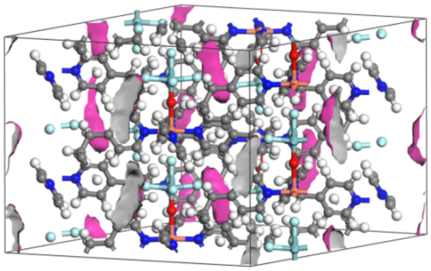 | 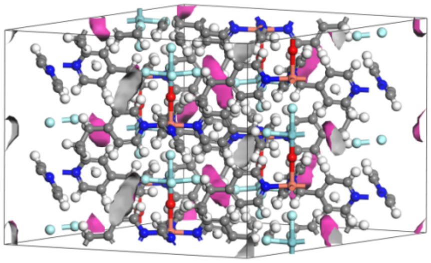 |
| **View 3** | 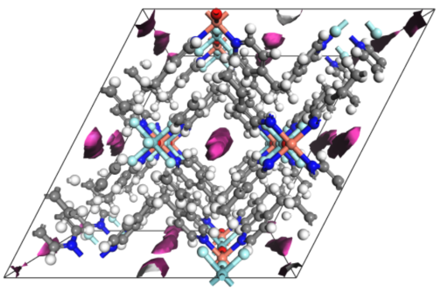 | 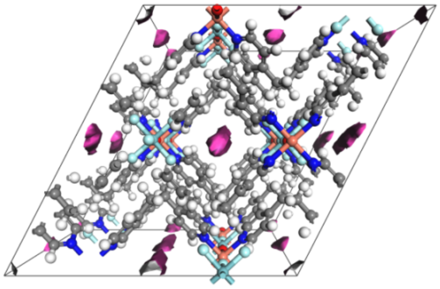 |

**Table S12.** Visualization from different view angles of $C_3H_4$ and $C_3H_6$ binding site isosurfaces from CMC simulations with 8 adsorbates in a 2x2x1 **sql-NbOFFIVE-bpe-Cu-AB-α** supercell.

| | **sql-NbOFFIVE-bpe-Cu-AB_$C_3H_4$** | **sql-NbOFFIVE-bpe-Cu-AB_$C_3H_6$** |
|---|---|---|

| | | |
|---|---|---|
| **View 1** | 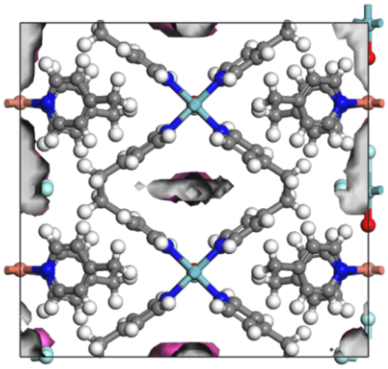 | 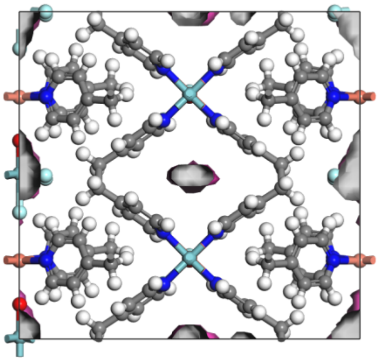 |
| **View 2** | 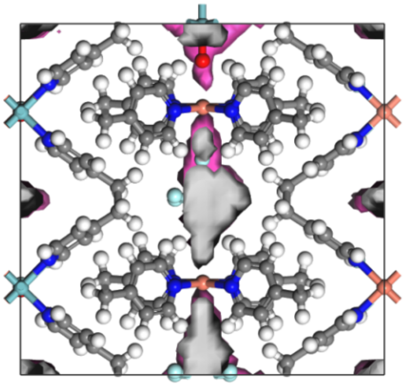 | 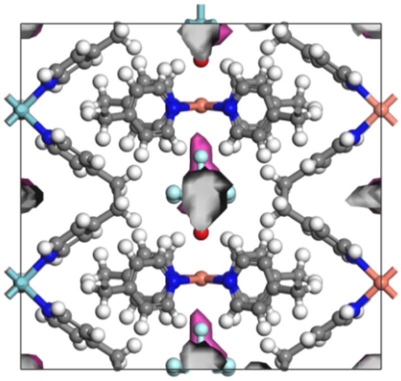 |
| **View 3** | 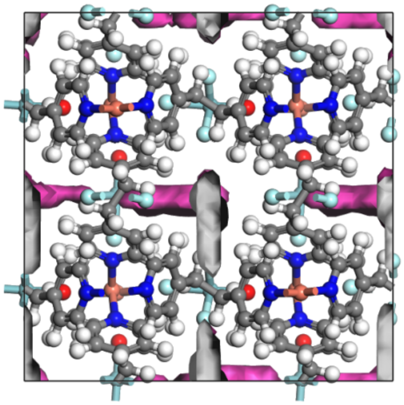 | 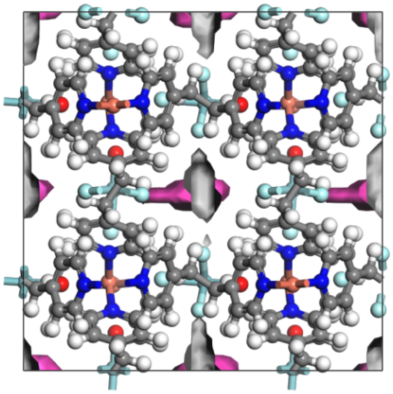 |
| **View 4** | 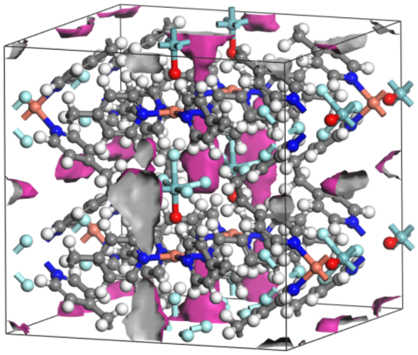 | 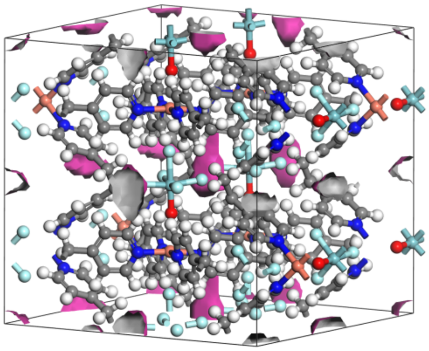 |

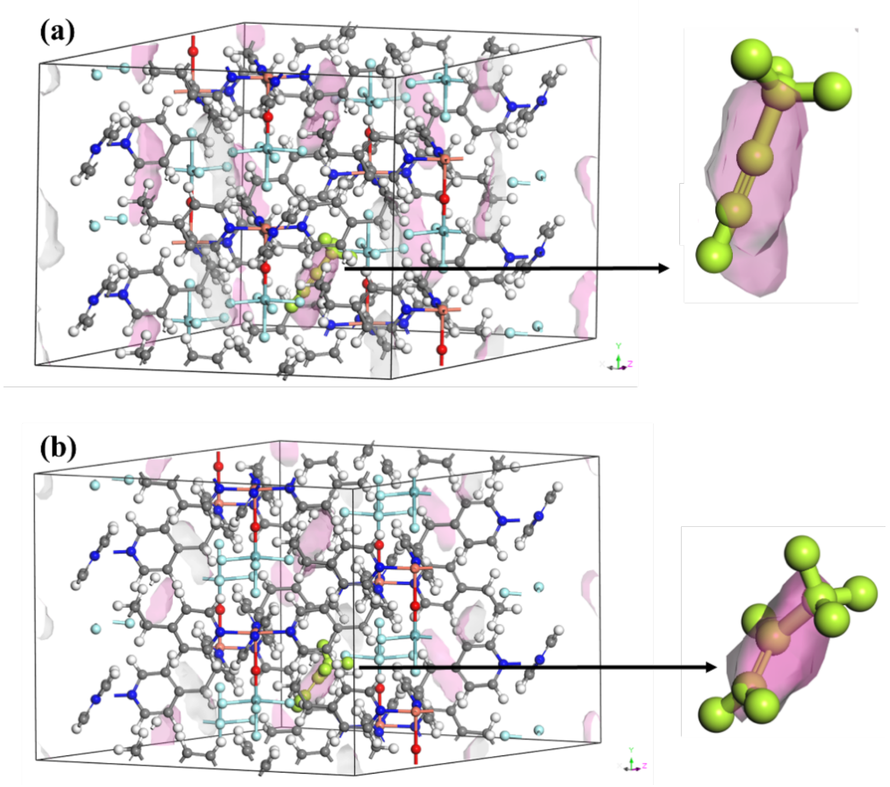


**Figure S31.** Visualization binding sites of (a) $C_3H_4$ and (b) $C_3H_6$ by CMC (pink isosurfaces) and DFT (light green ball and stick model) for **sql-NbOFFIVE-bpe-Cu-AA-α** framework.

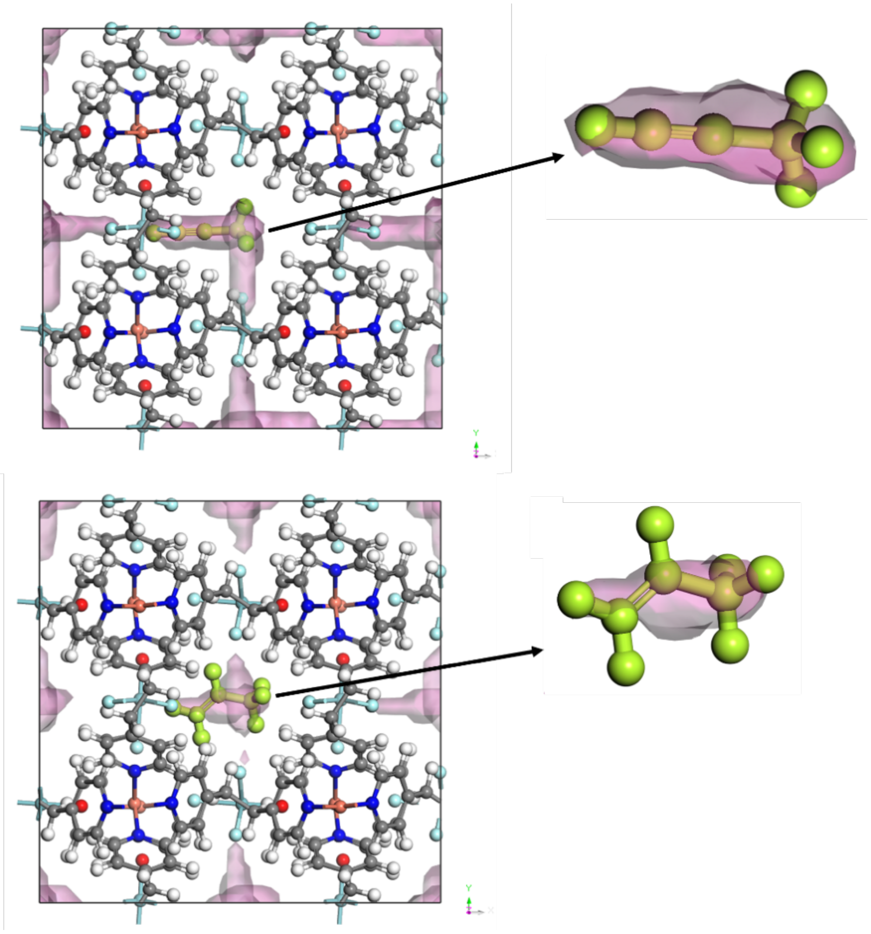

**Figure S32.** Visualization binding sites of (a) $C_3H_4$ and (b) $C_3H_6$ by CMC (pink isosurfaces) and DFT (light green ball and stick model) for **sql-NbOFFIVE-bpe-Cu-AB-α** framework.

# Notation

| | |
|---|---|
| $b$ | Langmuir-Freundlich constant, $\mathrm{Pa}^{-\nu}$ |
| $m_{ads}$ | mass of adsorbent in fixed bed, kg |
| $p$ | pressure, Pa |
| $q$ | component molar loading, mol $kg^{-1}$ |
| $q_{sat}$ | saturation loading, mol $kg^{-1}$ |

$Q_0$ volumetric flow rate of gas mixture at inlet to fixed bed, L $s^{-1}$

## Greek letters

$\nu$ Freundlich exponent, dimensionless

$\tau$ time, dimensionless

# References

[1] Bondi, A. van der Waals Volumes and Radii. *J. Phys. Chem. A.* **1964,** *68*, 441-451.

[2] Krause, L.; Herbst-Irmer, R.; Sheldrick, G. M.; Stalke, D. Comparison of silver and molybdenum microfocus x-ray sources for single-crystal structure determination. *J Appl Crystallogr.* **2015,** *48*, 3-10.

[3] Gannon, R. E.; Krukonis, V. J.; Schoenberg, T. Conversion of Coal to Acetylene in Arc-Heated Hydrogen. *Product R&D.* **1970,** *9*, 343-347.

[4] Sheldrick, G. SHELXT - Integrated space-group and crystal-structure determination. *Acta Crystallogr. A.* **2015,** *71*, 3-8.

[5] Sheldrick, G. Crystal structure refinement with SHELXL. *Acta Crystallogr. Sect. C.* **2015,** *71*, 3-8.

[6] Dolomanov, O. V.; Bourhis, L. J.; Gildea, R. J.; Howard, J. A. K.; Puschmann, H. OLEX2: a complete structure solution, refinement and analysis program. *J Appl Crystallogr.* **2009,** *42*, 339-341.

[7] Spackman, P. R.; Turner, M. J.; McKinnon, J. J.; Wolff, S. K.; Grimwood, D. J.; Jayatilaka, D.; Spackman, M. A. CrystalExplorer: a program for Hirshfeld surface analysis,

visualization and quantitative analysis of molecular crystals. *J. Appl. Crystallogr.* **2021,** *54*, 1006-1011.

[8] Peng, Y. L.; Wang, T.; Jin, C.; Li, P.; Suepaul, S.; Beemer, G.; Chen, Y.; Krishna, R.; Cheng, P.; Pham, T.; Space, B.; Zaworotko, M. J.; Zhang, Z. A robust heterometallic ultramicroporous MOF with ultrahigh selectivity for propyne/propylene separation. *J. Mater. Chem. A.* **2021,** *9*, 2850-2856.

[9] Gutierrez-Sevillano, J. J.; Calero, S.; Krishna, R. Separation of benzene from mixtures with water, methanol, ethanol, and acetone: highlighting hydrogen bonding and molecular clustering influences in CuBTC. *Phys. Chem. Chem. Phys.* **2015,** *17*, 20114-24.

[10] Gutiérrez-Sevillano, J. J.; Calero, S.; Krishna, R. Selective adsorption of water from mixtures with 1-alcohols by exploitation of molecular packing effects in CuBTC. *J. Phys. Chem. C.* **2015,** *119*, 3658-3666.

[11] PRAUSNITZ, A. L. M. a. J. M. Thermodynamics of mixed-gas adsorption. *AIChE. J.*. **1965,** *11*, 121-127.

[12] Krishna, R. The Maxwell-stefan description of mixture diffusion in nanoporous crystalline materials. *Microporous Mesoporous Mater.* **2014,** *185*, 30-50.

[13] Krishna, R. Methodologies for evaluation of metal-organic frameworks in separation applications. *RSC Adv.* **2015,** *5*, 52269-52295.

[14] Krishna, R. Screening Metal-Organic Frameworks for mixture separations in fixed-bed adsorbers using a combined selectivity/capacity metric. *RSC Adv.* **2017,** *7*, 35724-35737.

[15] Krishna, R. Methodologies for screening and selection of crystalline microporous materials in mixture separations. *Sep. Purif. Technol.* **2018,** *194*, 281-300.

[16] Krishna, R. Metrics for evaluation and screening of metal-organic frameworks for applications in mixture separations. *ACS Omega.* **2020,** *5*, 16987−17004.

[17] Yao, Z.; Zhang, Z.; Liu, L.; Li, Z.; Zhou, W.; Zhao, Y.; Han, Y.; Chen, B.; Krishna, R.; Xiang, S. Extraordinary separation of acetylene-containing mixtures with microporous metal-organic frameworks with open o donor sites and tunable robustness through control of the helical chain secondary building units. *Chem. Eur. J.* **2016,** *22*, 5676-5683.

[18] Chen, K. J.; Scott, Hayley S.; Madden, David G.; Pham, T.; Kumar, A.; Bajpai, A.; Lusi, M.; Forrest, Katherine A.; Space, B.; Perry, John J.; Zaworotko, Michael J. Benchmark $C_2H_2/CO_2$ and $CO_2/C_2H_2$ separation by two closely related hybrid ultramicroporous materials. *Chem.* **2016,** *1*, 753-765.

[19] Nugent, P.; Belmabkhout, Y.; Burd, S. D.; Cairns, A. J.; Luebke, R.; Forrest, K.; Pham, T.; Ma, S.; Space, B.; Wojtas, L.; Eddaoudi, M.; Zaworotko, M. J. Porous materials with optimal adsorption thermodynamics and kinetics for $CO_2$ separation. *Nature.* **2013,** *495*, 80-4.

[20] Cadiau, A.; Belmabkhout, Y.; Adil, K.; Bhatt, P. M.; Pillai, R. S.; Shkurenko, A.; Martineau-Corcos, C.; Maurin, G.; Eddaoudi, M. Hydrolytically stable fluorinated metal-organic frameworks for energy-efficient dehydration. *Science.* **2017,** *356*, 731-735.

[21] Mukherjee, S.; Kumar, N.; Bezrukov, A. A.; Tan, K.; Pham, T.; Forrest, K. A.; Oyekan, K. A.; Qazvini, O. T.; Madden, D. G.; Space, B.; Zaworotko, M. J. Amino-functionalised hybrid ultramicroporous materials that enable single-step ethylene purification from a ternary mixture. *Angew. Chem. Int. Ed.* **2021,** *60*, 10902-10909.

[22] Subramanian, S.; Zaworotko, M. J. Porous Solids by Design : $[Zn(4,4'\text{-bpy})_2(SiF_6)_n\cdot x$ DMF, a single framework octahedral coordination polymer with large square channel. *Angew. Chem. Int. Ed.* **1995,** *34*, 2127-2128.

[23] Lin, M. J.; Jouaiti, A.; Kyritsakas, N.; Hosseini, M. W. Molecular tectonics: control of interpenetration in cuboid 3-D coordination networks. *CrystEngComm.* **2011,** *13*, 776-778.

[24] Suen, M. C.; Chan, Z. K.; Chen, J. D.; Wang, J. C.; Hung, C.-H. Syntheses and structures of three new coordination polymers generated from the flexible 1,3-bis(4-pyridyl)propane ligand and zinc salts. *Polyhedron.* **2006,** *25*, 2325-2332.

[25] Lin, M. J.; Jouaiti, A.; Pocic, D.; Kyritsakas, N.; Planeix, J. M.; Hosseini, M. W. Molecular tectonics: tubular crystals with controllable channel size and orientation. *Chem. Commun.* **2010,** *46*, 112-4.

[26] Lin, R. B.; Li, L. B.; Wu, H.; Arman, H.; Li, B.; Lin, R. G.; Zhou, W.; Chen, B. L. Optimized separation of acetylene from carbon dioxide and ethylene in a microporous material. *J. Am. Chem. Soc.* **2017,** *139*, 8022-8028.

[27] Wang, J.; Zhang, Y.; Zhang, P.; Hu, J.; Lin, R. B.; Deng, Q.; Zeng, Z.; Xing, H.; Deng, S.; Chen, B. Optimizing pore space for flexible-robust Metal-Organic Framework to boost trace acetylene removal. *J. Am. Chem. Soc.* **2020,** *142*, 9744-9751.

[28] Ke, T.; Wang, Q.; Shen, J.; Zhou, J.; Bao, Z.; Yang, Q.; Ren, Q. Molecular sieving of C2 -C3 alkene from alkyne with tuned threshold pressure in robust layered Metal-Organic Frameworks. *Angew. Chem. Int. Ed.* **2020,** *59*, 12725-12730.

[29] Shen, J.; He, X.; Ke, T.; Krishna, R.; van Baten, J. M.; Chen, R.; Bao, Z.; Xing, H.; Dinca, M.; Zhang, Z.; Yang, Q.; Ren, Q. Simultaneous interlayer and intralayer space control in two-dimensional metal-organic frameworks for acetylene/ethylene separation. *Nat Commun.* **2020,** *11*, 6259.

[30] M. Shivanna, K. I. Otake, B. Q. Song, L. M. van Wyk, Q. Y. Yang, N. Kumar, W. K. Feldmann, T. Pham, S. Suepaul, B. Space, L. J. Barbour, S. Kitagawa, M. Zaworotko.

Benchmark acetylene binding affinity and separation through induced fit in a flexible hybrid ultramicroporous material. *Angew. Chem. Int. Ed.* **2021**, *60*, 20383–20390.

[31] Wang, J.; Zhang, Y.; Su, Y.; Liu, X.; Zhang, P.; Lin, R. B.; Chen, S.; Deng, Q.; Zeng, Z.; Deng, S.; Chen, B. Fine pore engineering in a series of isoreticular metal-organic frameworks for efficient $C_2H_2/CO_2$ separation. *Nat. Commun.* **2022,** *13*, 200.

[32] Kumar, N.; Mukherjee, S.; Bezrukov, A. A.; Vandichel, M.; Shivanna, M.; Sensharma, D.; Bajpai, A.; Gascón, V.; Otake, K. i.; Kitagawa, S.; Zaworotko, M. J. A square lattice topology coordination network that exhibits highly selective $C_2H_2/CO_2$ separation performance. *SmartMat.* **2020,** *1*, e1008.

[33] Blochl, P. E. Projector augmented-wave method. *Phys Rev B* **1994,** *50*, 17953-17979.

[34] Kresse, G.; Furthmuller, J. Efficiency of ab-initio total energy calculations for metals and semiconductors using a plane-wave basis set. *Comput. Mater. Sci* **1996,** *6*, 15-50.

[35] Kresse, G.; Furthmuller, J. Efficient iterative schemes for ab initio total-energy calculations using a plane-wave basis set. *Phys Rev B Condens Matter* **1996,** *54*, 11169-11186.

[36] Wellendorff, J.; Lundgaard, K. T.; Møgelhøj, A.; Petzold, V.; Landis, D. D.; Nørskov, J. K.; Bligaard, T.; Jacobsen, K. W. Density functionals for surface science: Exchange-correlation model development with Bayesian error estimation. *Physical Review B* **2012,** *85*, 235149

[37] Ghysels, A.; Verstraelen, T.; Hemelsoet, K.; Waroquier, M.; Van Speybroeck, V. TAMkin: a versatile package for vibrational analysis and chemical kinetics. *J. Chem. Inf. Model.* **2010,** *50*, 1736-1750.

[38] Frenkel, D.; Smit, B. Understanding Molecular Simulation: From Algorithms to Applications. *Academic Press: New York* **2002**, 112-114.

[39] Dubbeldam, D.; Calero, S.; Ellis, D. E.; Snurr, R. Q. RASPA: molecular simulation software for adsorption and diffusion in flexible nanoporous materials. *Mol. Simul.* **2016,** *42*, 81-101.

[40] Dubbeldam, D.; Torres-Knoop, A.; Walton, K. S. On the inner workings of Monte Carlo codes. *Mol. Simul.* **2013,** *39*, 1253-1292.

[41] BIOVIA, Dassault Systèmes, Material Studio, San Diego: Dassault Systèmes, 2022.

[42] Wilmer, C. E.; Kim, K. C.; Snurr, R. Q. An Extended Charge Equilibration Method. *J Phys Chem Lett* **2012,** *3*, 2506-2511.

[43] Rappe, A. K.; Casewit, C. J.; Colwell, K. S.; Goddard, W. A.; Skiff, W. M. Uff, a Full Periodic-Table Force-Field for Molecular Mechanics and Molecular-Dynamics Simulations. *J. Am. Chem. Soc.* **1992,** *114*, 10024-10035.

[44] Boyd, P. G.; Moosavi, S. M.; Witman, M.; Smit, B. Force-Field Prediction of Materials Properties in Metal-Organic Frameworks. *J Phys Chem Lett* **2017,** *8*, 357-363.
